# Synthetic Efforts toward the Synthesis of a Fluorinated Analog of 5-Aminolevulinic Acid: Practical Synthesis of Racemic and Enantiomerically Defined 3-Fluoro-5-aminolevulinic Acid

**DOI:** 10.1021/acs.joc.4c01070

**Published:** 2024-08-27

**Authors:** Gouthami Pashikanti, Lahu N. Chavan, Lanny S. Liebeskind, Mark M. Goodman

**Affiliations:** † Department of Radiology and Imaging Sciences, School of Medicine, Emory University, 1364 Clifton Road NE, Atlanta, Georgia 30322, United States; ‡ Department of Chemistry, Emory University, 1515 Dickey Drive, Atlanta, Georgia 30322, United States; § Center for Systems Imaging, Emory University, 1841 Clifton Rd NE, Atlanta, Georgia 30322, United States

## Abstract

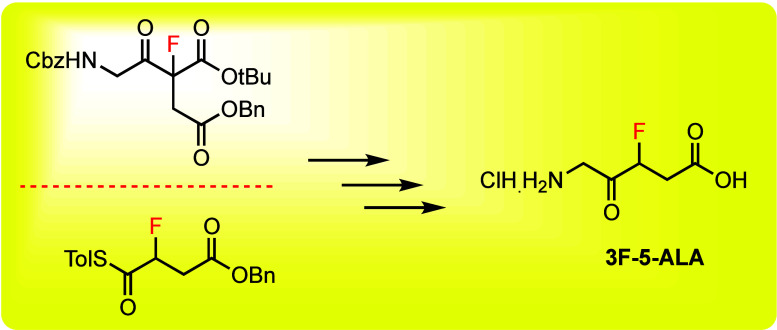

In 2017, the FDA authorized 5-aminolevulinic acid (5-ALA) for intraoperative optical imaging of suspected high-grade gliomas. This was the first authorized optical imaging agent for brain tumor surgery to enhance the visualization of malignant tissue. Herein we report the synthesis of a racemic and enantiopure fluorinated analog of 5-ALA, i.e., 3-fluoro-5-aminolevulinic acid (3F-5-ALA). We anticipate that these studies will provide the foundation for the future construction of a fluorine-18-labeled 5-ALA PET tracer to be used for functional and metabolic imaging of gliomas.

## Introduction

Brain tumors represent a prominent category of severe and life-threatening malignancies on a global scale. Within this category, gliomas emerge as the most prevalent primary neoplasms within the central nervous system (CNS), constituting approximately 80% of malignant brain tumors.^1^ Their molecular and cellular heterogeneity promotes proliferation, invasion, and treatment resistance.^2^ Therefore, noninvasive imaging technologies that reveal a tumor’s molecular and metabolic information are needed for subtype-specific and individualized treatment for gliomas. Despite the fact that magnetic resonance imaging (MRI) is the clinical gold standard for imaging gliomas due to its superior spatial resolution and soft tissue contrast, its molecular and metabolic imaging applications suffer from low sensitivity.^3^ In contrast, positron emission tomography (PET) appears promising and offers insight into the molecular biology of tissues across the body.^4^ However, the currently used fluorodeoxyglucose (FDG) PET has low specificity and is unsuitable for imaging brain tumors due to significant FDG uptake in normal brain tissue.^5a,5b^ In contrast, [^11^C]methionine is valuable for PET imaging in oncology, particularly for gliomas, due to its ability to trace tumor metabolism, but its short half-life poses logistical challenges.^5c^ Currently, there is a significant unmet demand for highly diverse and infiltrating molecular imaging probes for gliomas.

5-Aminolevulinic acid (5-ALA) is the first natural precursor metabolite in the heme biosynthesis pathway; it is typically produced from succinyl-CoA and alanine.^6^ Protoporphyrin IX (PPIX) is a fluorescent red molecule that represents the final metabolite before heme production.^6^ During the synthesis of PpIX, 5-ALA is the rate-limiting intermediate step, and oral administration of 5-ALA causes selective accumulation of 5-ALA and PpIX within the pathological tissue, particularly Glioblastomas (GBMs).^7^ Its preferential accumulation within glioma cells is associated with decreased amounts of the ferrochelatase enzyme and impaired cellular clearance by an ATP-binding cassette transporter (ABCB6).^8^ It has been demonstrated that 5-ALA-induced PpIX fluorescence is highly selective (98%) for neoplastic tissue in patients with known glioblastoma multiforme.^9^ Exogenous administration of 5-ALA causes selective accumulation of 5-ALA and PpIX in gliomas, enabling tumor cell-specific intraoperative identification of the tumor margin for tumor resection with optical devices and photodynamic treatment of high grade gliomas (HGGs),^10^ both of which have been FDA-approved for the treatment of gliomas. Even though ALA-mediated PDT offers several benefits, it has been utilized mostly for superficial lesions due to poor light penetration within the tissue.^11,12^ The combination of PET imaging and a radiolabeled form of 5-ALA can overcome the constraints of optical detectability and provide insight into diagnostic and therapeutic techniques (for presurgical planning purposes) with 3D rendering capacity at the molecular level throughout the body.

**Figure 1:**
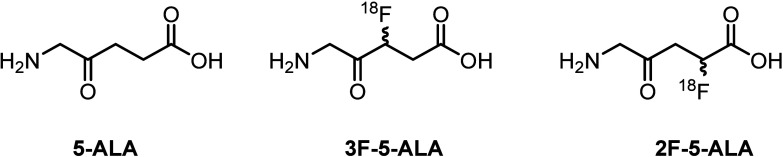
5-Aminolevulinic acid (5-ALA) as the lead compound for ^18^F PET radiotracer development

The possible PET radioisotopes suitable for 5-ALA are ^11^C, ^13^N, and ^15^O. To date, Goodman and co-workers have successfully synthesized [^13^N] 5-ALA in high radiochemical yield (65%) and validated the rapid tumor-specific uptake of [^13^N] 5-ALA in rats bearing intracranial 9L glioblastoma.^13^ However, the extremely short half-life of the ^13^N tracer limits its use for further applications. The longer half-life of ^18^F compared to those of ^13^N and ^11^C (110.8 min for ^18^F vs 9.98 min for ^13^N and 20.3 min for ^11^C) makes it the ideal radionuclide for therapeutic PET imaging; hence, we envisioned producing a fluorine-18 derivative of 5-ALA. In order to develop a stable ^18^F PET radioligand of 5-ALA, fluorine-18 can be introduced by substituting hydrogen at the 2- or 3-carbon positions of the ALA core, as illustrated in Figure 1. There have been reports of nonradioactive forms of 2-fluoro-5-ALA (2F-5-ALA) and 2,2′-difluoro-5-ALA.^14^ Since 2F-5-ALA is an inhibitor of 5-aminolevulinate dehydratase (*K*_i_ = 0.5–2 mM), it can only be used to determine the production rate of PpIX and is not suitable for PET imaging. Based on the inhibitory effect of 2F-ALA to aminolevulinate dehydratase, we chose to define and synthesize a nonradioactive version of 3-fluoro-5-ALA (3F-5-ALA) and evaluate its optical characteristics to determine if fluorination at 3-position affects glioma cell uptake and metabolism to produce PpIX. In this manuscript, we disclose our synthetic efforts for constructing 3-fluoro-5-ALA and its enantiomers as a prelude to future disclosures of 18F analogs of 5-ALA.

## Results and Discussion

**Scheme 1:**
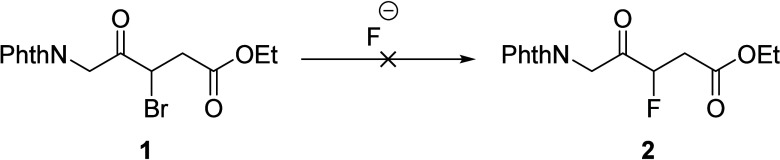
Attempts to Fluorinate Levulinate **1**

Our first approach to 3-fluorinated 5-ALA began with the substitution of the bromine atom of the requisite functionalized levulinate **1** (prepared by a known protocol^15^) with nucleophilic fluoride (Scheme 1). However, many attempts to fluorinate bromide **1** under the required basic conditions, including (CsF/*t*BuOH, KF/18-crown-6/ACN, TBAF/THF, etc.) were thwarted by the formation of the olefinic byproduct via elimination of HBr, undoubtedly facilitated by the presence of the ester group.

**Scheme 2:**
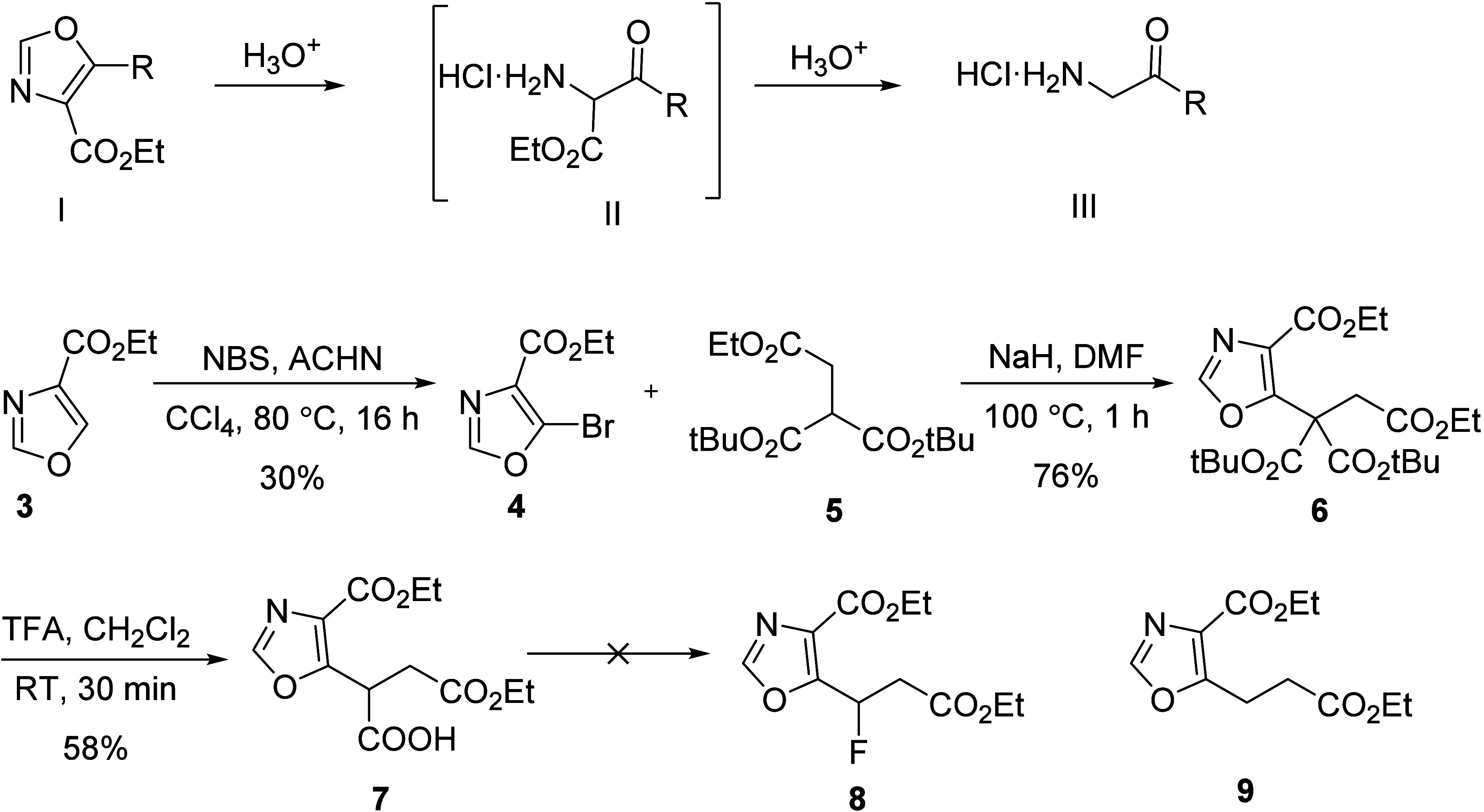
Decarboxylative Fluorination Approach

Based on these initial observations, we concluded that the oxo and carboxylic acid functionalities of 3-F-5-ALA should be revealed later in the synthetic scheme to favor S_N_2 formation of the desired fluorinated product over the competitive β-elimination product since these activating groups enhance the formation of a double bond. We thus sought out appropriate protecting groups for the oxo and carboxylic acid groups that could be exposed post fluorination. After an extensive investigation, we were led to protectively embed both the amino and oxo functionalities of 3F-5-ALA within an oxazole precursor^16^ as depicted at the top of Scheme 2 (I–II–II). We anticipated a gentle fluorodecarboxylation^17^ to introduce the fluorine atom (bottom of Scheme 2) followed by a hydrolytic reveal of the 5-ALA skeleton.

Thus, we began our plans with the construction of a functionalized derivative of oxazole that would be amenable to decarboxylative fluorination (**7** in Scheme 2). In accordance with the existing literature, commercially available ethyl oxazole-4-carboxylate **3** was selectively brominated to give ethyl 5-bromooxazole-4-carboxylate **4**.^18^ This underwent S_N_Ar displacement with the sodium enolate of malonate **5**, yielding the triester intermediate **6** in 76% yield. One-pot hydrolysis of the tertiary butyl esters of **6** followed by decarboxylation to the monoacid **7** proved challenging due to overdecarboxylation resulting in the formation of **9**. Fortunately, when compound **6** was treated with a 1:1 TFA/CH_2_Cl_2_ mixture for 30 min at room temp, monoacid **7** was produced in 58% yield (Scheme 2).

With the requisite monoacid **7** in hand, we focused on the crucial decarboxylative fluorination reaction. Unfortunately, numerous attempts to fluorinate **7** with electrophilic^17a,17b^ and nucleophilic fluorinating^17c^ agents were futile, all resulting in the formation of the decarboxylative product **9**. We postulated that the electron-deficient oxazole ring might activate the benzylic carboxylic acid **7** toward decarboxylation leading to **9**.

**Scheme 3:**
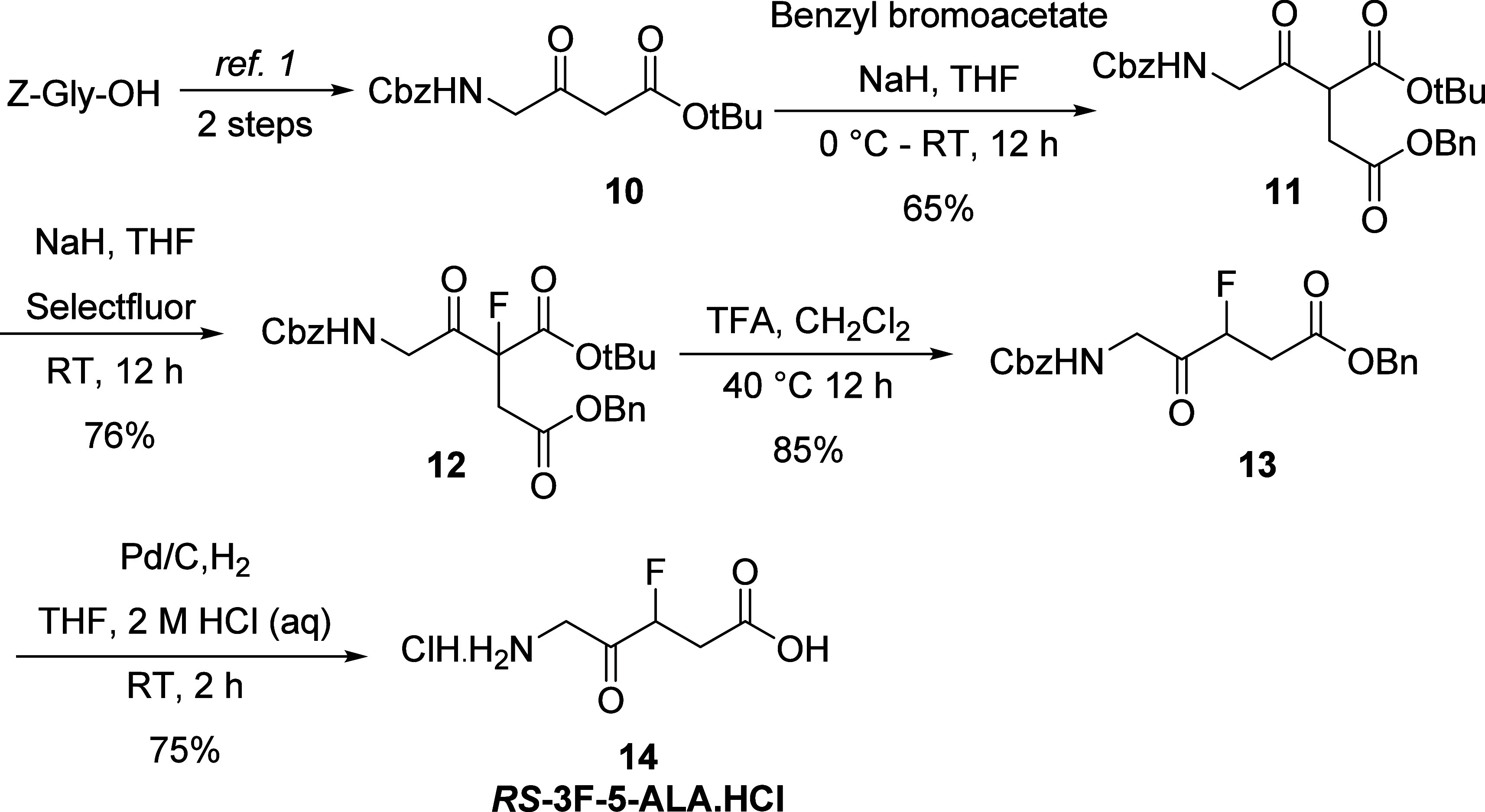
Synthesis of *rac*-3F-5-ALA

As an alternative, a straightforward approach for synthesizing *rac*-3F-5-ALA using electrophilic fluorination of intermediate **11** with Selectfluor followed by decarboxylation under acidic conditions is depicted in Scheme 3. *N*-Cbz γ-amino-β-keto ester **10** (Scheme 3) is readily accessible in two steps from commercially available Cbz-glycine.^19^ Treatment of *N*-Cbz γ-amino-β-keto ester **10** with NaH and benzyl bromoacetate in tetrahydrofuran led to the formation of keto diester **11** in 62% yield along with the recovery of starting material. Keto diester **11** was fluorinated using Selectfluor in the presence of sodium hydride (76% yield). The resulting product was then refluxed with trifluoroacetic acid in dichloromethane to afford monofluoroketoester **13** in 85% yield. Finally, hydrogenation of the monofluoroketoester **13** in a mixture of THF/aq. 2 M HCl (4:1) solvent system furnished the desired *rac*-3-fluoro-5-aminolevulinic acid hydrochloride (3-F-5-ALA·HCl) **14** in 75% yield.

**Scheme 4:**
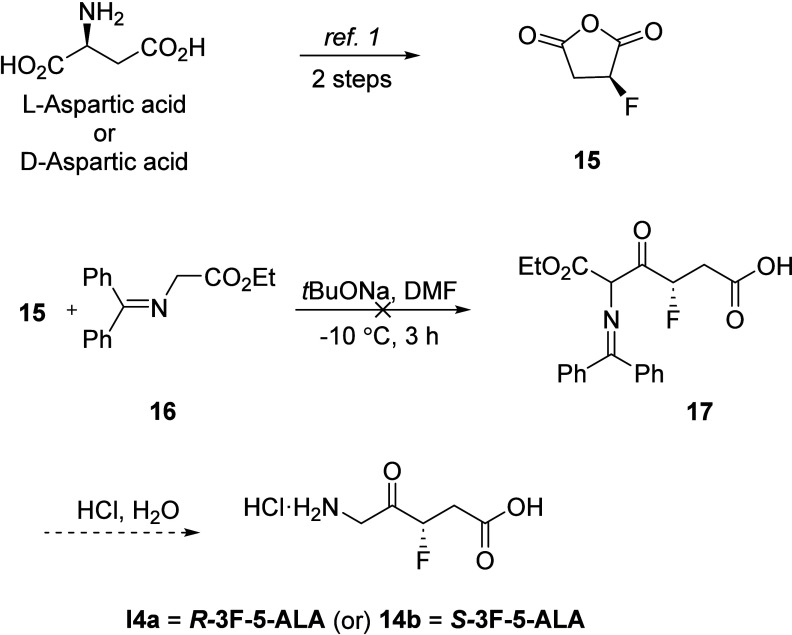
Synthesis of 3F-5-ALA Enantiomers from Fluorosuccinic Anhydride **15**

Having established a successful route for the synthesis of racemic 3F-5-ALA, we then turned our attention to the construction of enantiopure 3F-5-ALA. A literature search revealed four examples describing the synthesis of 5-ALA by ring-opening of succinic anhydride with different nucleophiles (*N*,*N*-diphenylmethylidene glycine ethyl ester, KCN, ethyl hippurate, and methyl nitro acetate).^20^ Based on this precedent, it was anticipated that both enantiomers of 3F-5-ALA would be accessible via the regioselective ring-opening of the appropriate enantiomeric 2-fluorosuccinic anhydride **15** with *N*,*N*-diphenylmethylidene glycine ethyl ester in the presence of a base, followed by acidic hydrolysis to give 3F-5-ALA·HCl (**14a** or **14b**), as depicted in Scheme 4.

The synthesis started with the preparation of *S*-fluorosuccinic anhydride **15** (Scheme 4), which is readily accessible from l-aspartic acid, in two steps.^21^ Unfortunately, all attempts reacting **15** with any of the nucleophiles^20^ mentioned earlier, including the use of different solvent systems and moderating additives (MgBr_2_ or ZnCl_2_), did not provide any discrete ring-opening products.

**Scheme 5:**
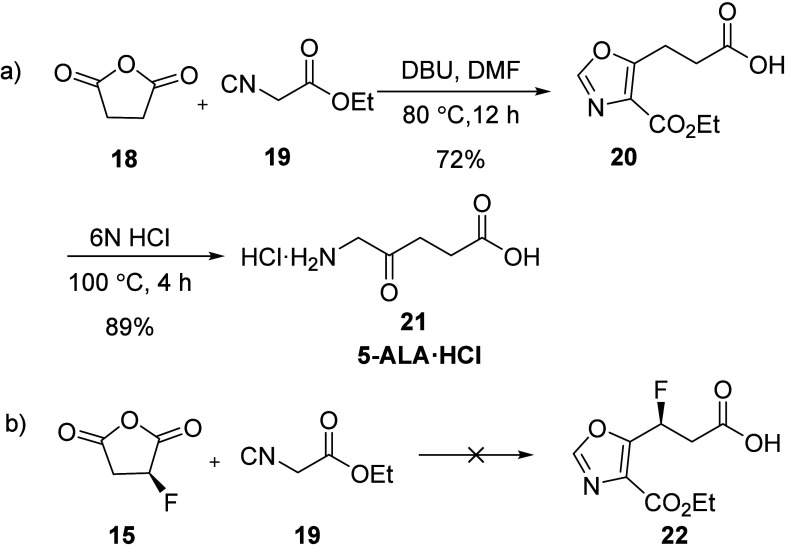
A New Approach to the Synthesis of 5-ALA

We next examined a model reaction adding ethyl isocyanate to succinic anhydride following Wilfred’s strategy,^22^ as depicted in Scheme 5. Interestingly, an oxazole intermediate was formed, which after hydrolysis with 6 N HCl, afforded 5-ALA in 64% yield for two steps (Scheme 5a). Thus, this set of reactions provides a new, high-yield synthesis of 5-ALA·HCl **21** from readily available, inexpensive starting materials. Unfortunately, fluorosuccinic anhydride **15** did not survive under the reaction conditions (Scheme 5b).

**Scheme 6:**
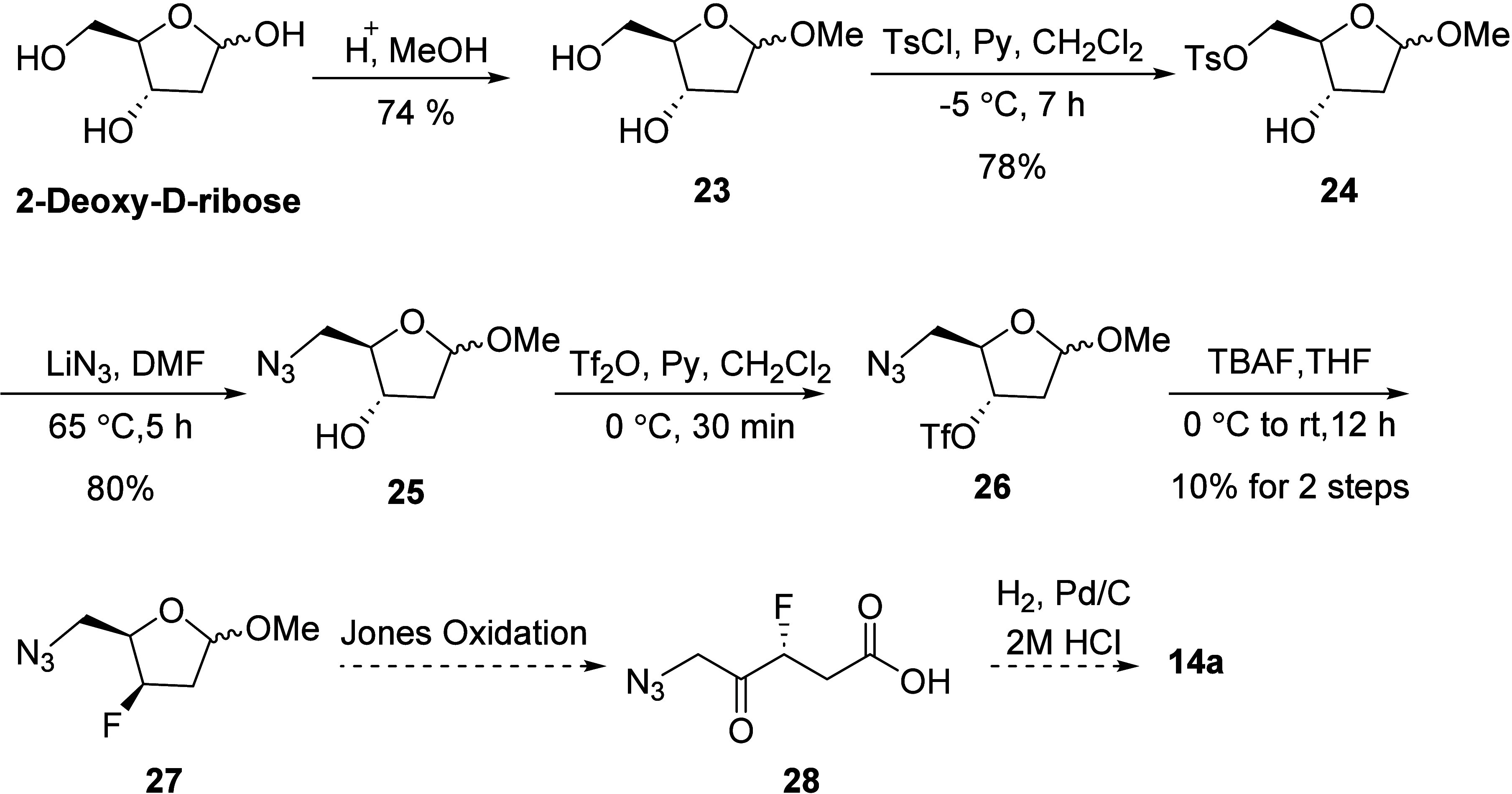
Proposed Scheme for the Synthesis of **14a** from 2-Deoxy-d-ribose

Next, a different strategy for the synthesis of *R*- and *S*-3F-5-ALA was envisaged from 2-deoxyribose, as shown in Scheme 6. In this approach (the Scheme shows only one of the enantiomers), the enantiomers of 3F-5-ALA would be obtained by overoxidation of **27** using Jones’ reagent followed by reduction of the azide to the amine in an acidic medium (Scheme 6). The precursor **27** would be derived from the nucleophilic fluorination of **26**, the latter produced from 2-deoxyribose in four steps.^23^ As in our previous strategy, the addition of various enolates to fluorosuccinic anhydride proved difficult (Scheme 4). However, contrary to the previous approach, protecting the 2-deoxyribose as cyclic acetal did prevent the elimination and provided the necessary framework.

As outlined in Scheme 6, the new synthesis commenced with the preparation of the known intermediate **25** in three steps. Thus, following the protocol detailed by Gottschaldt,^23^ commercially available 2-deoxy-d-ribose was treated with catalytic amounts of sulfuric acid in dry methanol, and furnished the cyclic acetal **23** (74%) as a mixture of α+β anomers. In practice, both anomers were utilized since the anomeric center is oxidized later in the synthesis. Conversion of compound **23** into the corresponding 5-O-tosyl-intermediate **24** (78%) was readily achieved under standard conditions, and this was then treated with lithium azide in DMF to afford the 5-azido-derivative **25** in 80% yield.^23^

**Scheme 7:**
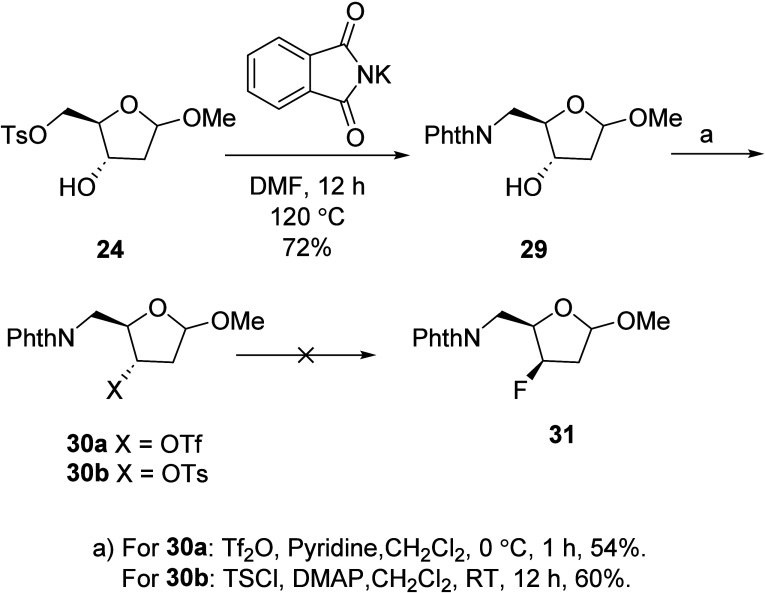
Attempts to Fluorinate Amino Ribose Derivatives **30a** and **30b**

Subsequent treatment of **25** with triflic anhydride in CH_2_Cl_2_ in the presence of pyridine at 0 °C afforded the highly unstable triflate **26**, which was used without purification in the next reaction. Attempts to fluorinate triflate **26** using CsF/*t*BuOH^24^ or KF/18-crown-6^25^ were unsuccessful, instead leading to the formation of unidentified products. However, reaction of the triflate **25** with TBAF/THF did produce the required product **27** but never in yields better than 10% (Scheme 6). Although we were able to access the fluorinated cyclic acetal **27** using this route, the poor reaction yield precluded its viability. We therefore investigated amino ribose derivatives **30a** toward nucleophilic fluorination followed by oxidation, as detailed in Scheme 7.

Thus, tosylate **24** was treated with potassium phthalimide to produce the protected amino ribose derivative **29** (Scheme 7), which was then treated with triflic anhydride to produce the unstable triflate **30a**, which could not be purified. Efforts to replace the triflate moiety of **30a** with fluoride led to the formation of olefinic byproducts. We hypothesized that a more stable leaving group might survive under the reaction conditions and produce the desired product. Consequently, compound **29** was treated with TsCl in the presence of DMAP to produce a stable tosylate **30b** in 60% yield (Scheme 7). Then, to obtain the desired fluorinated product **31**, we explored the conversion of **30b** under various nucleophilic fluorination conditions (CsF/*t*BuOH, TBAF/ACN at 80 °C and CsF/DMSO and KF/18-crown-6 in ACN 100 °C), but the reaction resulted in either recovery of **30b** or formation of elimination byproducts.

**Scheme 8:**
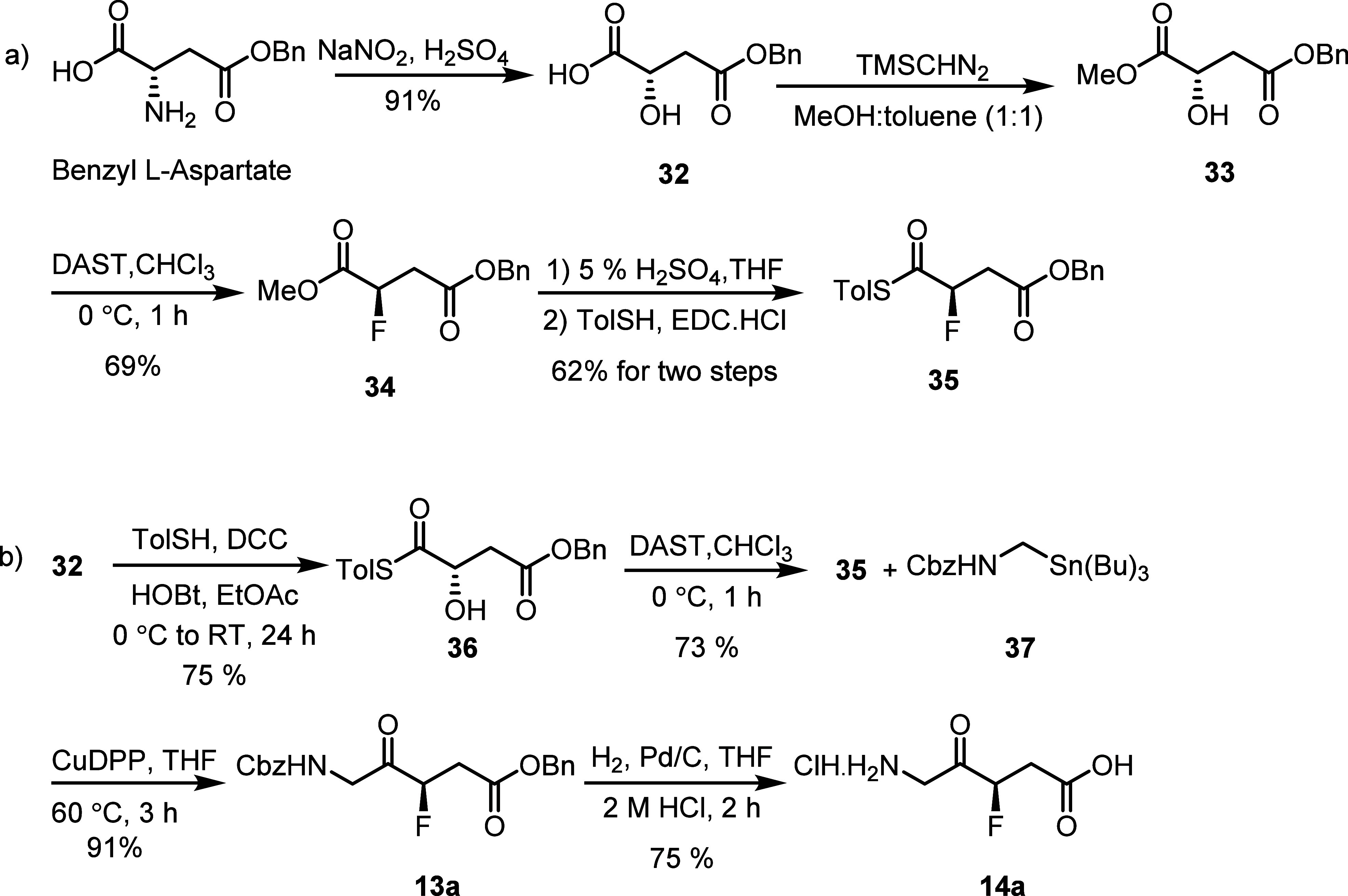
Successful Synthesis of *R*- and *S*-3F-5-ALA derivatives (**14a** and **14b**)

Once again, we needed to turn to an alternative synthetic strategy. At this point, it was clear that elimination to olefinic products under the basic nucleophilic fluorination conditions was the most significant obstacle to achieving our synthetic goal. Consequently, we focused on developing an efficient alternative strategy for synthesizing enantiopure 3F-5-ALA derivatives by maintaining strictly neutral or slightly acidic reaction conditions. We thus turned our attention to construction of the ketonic moiety of 5-ALA using the pH-neutral reaction conditions of the Liebeskind–Srogl cross-coupling reaction as shown in Scheme 8.^26,27^

To explore this potential, we conducted reactions using fluoro thioester **35** with alpha aminostannane **37** (Scheme 8b). The required ketonic precursor **36** was prepared from the known alcohol **33**, which can be obtained from commercially available benzyl l- (or d-) aspartate according to a literature procedure^28^ (Scheme 8a).

Fluorination of compound **33** in dichloromethane at −20 °C with DAST resulted in the formation of required product **34** in 48% yield along with the dehydrated olefinic product. However, switching the solvent from dichloromethane to chloroform and maintaining the reaction temperature at 0 °C throughout resulted in a significant improvement in the yield to 69%. Thus, compound **34** was synthesized in 3 steps with one chromatographic purification in 63% overall yield. Substrate **34** was subsequently converted into thioester **35** in a two-step sequence involving hydrolysis of the methyl ester and coupling the resulting acid group with 4-methylthiophenol using EDC·HCl (Scheme 8a). While we attained thioester in 39% overall yield over 5 steps, protection and deprotection of the acid in Scheme 8a were considered inefficient and unnecessary.

**Figure 2:**
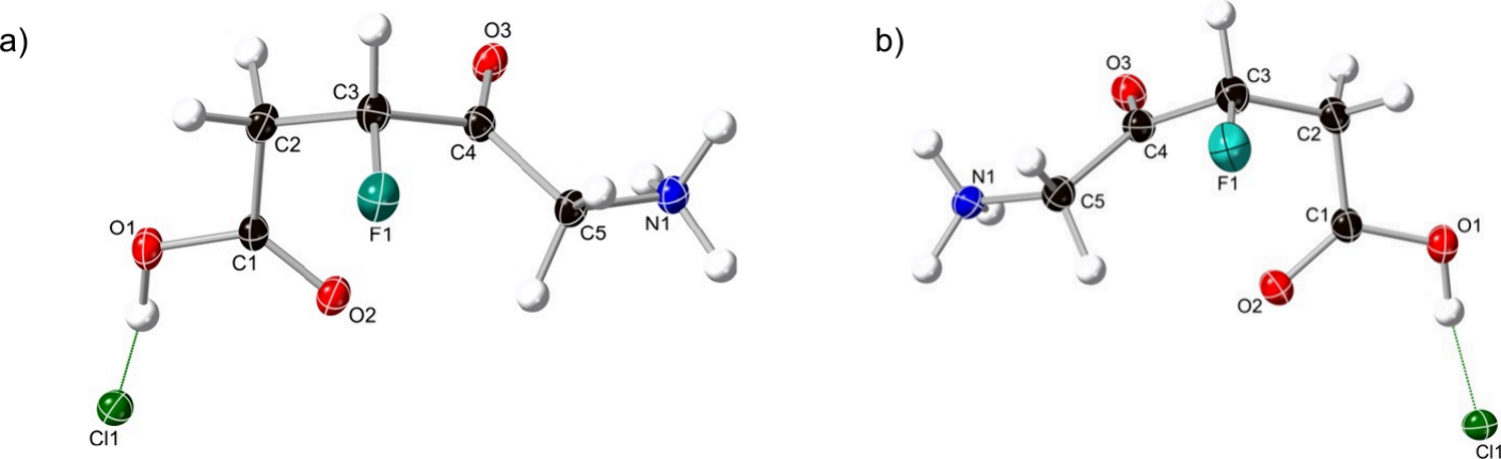
X-ray crystal structures of (a) *R*- and (b) *S*-3-F-5-ALA derivatives. Atom labels are as follows: white = hydrogen, black = carbon, red = oxygen, blue = nitrogen, light green = fluorine, and dark green = chlorine.

Therefore, we slightly modified the scheme by directly coupling carboxylic acid **32** and 4-methylthiophenol using DCC to furnish the thioester **36** in 75% yield (Scheme 8b). Treatment of alcohol **36** with DAST led to the formation of the fluoro thioester **35** (73%) with inversion of configuration (confirmed by X-ray crystallography as shown below). Benzyl tri-*n*-butylstannylmethylcarbamate **37** was synthesized from tri-*n*-butylstannylmethyl iodide in three steps following the literature precedent.^29^ Then, a pH-neutral Liebeskind-Srogl cross-coupling using 2 equiv of [copper(I) diphenylphosphinate (CuDPP, 2 equiv)^27^ in THF at 50 °C provided the desired ketonic coupling product **13a** in 91% yield. Compound **13a** was then subjected to deprotection hydrogenolysis in an acidic medium to furnish the desired *R*-3-F-5-ALA **14a** in 75% yield. Separately, *S*-3F-5-ALA **14b** was synthesized from benzyl d-aspartate using the same synthetic Scheme 8. The stereochemistry of the *R*- and *S*-3-F-5-ALA isomers was assigned by X-ray crystallography, as shown in Figure 2.

## Conclusion

This study reveals novel and scalable synthetic methods for producing racemic and enantiopure 3-fluoro-5-aminolevulinic acid hydrochloride (3F-5-ALA). The synthesis of racemic 3F-5-ALA·HCl (**14**) involves a six-step process utilizing electrophilic fluorination, resulting in a satisfactory overall yield of 16% from *N*-benzyloxycarbonylglycine (Z-Gly-OH). Additionally, both enantiomers of 3-F-ALA·HCl (**14a** and **14b**) were successfully constructed in a five-step procedure starting from benzyl L and d-aspartate, respectively, using a Liebeskind–Srogl cross-coupling as the crucial step. Notably, this enantioselective synthesis provided an overall yield of 34%. Moreover, we demonstrate a scalable approach for synthesizing 5-ALA from widely accessible, inexpensive starting materials in 2 steps in 64% yield. The biological evaluation of the 3-F-5-ALA derivatives will be pursued separately and published elsewhere.

## Experimental Section

### General Information

Unless otherwise noted, all reagents were used as received from commercial suppliers. All reactions were performed under a nitrogen atmosphere and in flame-dried or oven-dried glassware with magnetic stirring. All solvents were dried before use, following the standard procedures. Reactions were monitored using thin-layer chromatography (SiO_2_). TLC plates were visualized with UV light (254 nm), iodine treatment, or ninhydrin stain. Column chromatography was carried out using silica gel (60–120 mesh and 100–200 mesh) packed in glass columns. NMR spectra were recorded at 300, 400, 500 MHz (^1^H) and at 75, 100, 125 MHz (^13^C), respectively. Chemical shifts (δ) are reported in ppm, using the residual solvent peak in CDCl_3_ (^1^H, δ = 7.26; ^13^C, δ = 77.16 ppm) as internal standard, and coupling constants (*J*) are given in Hz. HRMS were recorded using ESI-TOF techniques.

### 1,1-Di-*tert*-butyl 2-Ethyl 1-(4-(Ethoxycarbonyl)oxazol-5-yl)ethane-1,1,2-tricarboxylate (**6**)

A suspension of sodium hydride (65.3 mg, 2.73 mmol, 2 equiv) in anhydrous DMF (1 mL) was stirred and treated dropwise at room temperature with a solution of diethyl malonate **5** (824 mg, 2.73 mmol, 2 equiv) in anhydrous DMF (2 mL). After 10 min, when gas evolution had ceased, the mixture was treated dropwise with a solution of oxazole **4** (300 mg, 1.36 mmol, 1 equiv) in anhydrous DMF (2 mL). The mixture turned orange and was stirred and heated at 100 °C in an oil bath for 1 h. The mixture was diluted with water and extracted with EtOAc (2 × 10 mL). The organics were then dried over anhydrous Na_2_SO_4_ and concentrated *in vacuo* to afford a yellow oil. The residue was chromatographed on silica, eluting with 10% EtOAc in hexanes (*R*_f_ = 0.5) to give the title compound **6** (457 mg, 76%) as a colorless oil. ^1^H NMR (600 MHz, CDCl_3_) δ 8.22 (s, 1H), 4.34 (q, *J* = 7.2 Hz, 2H), 4.16 (q, *J* = 7.2 Hz, 2H), 3.36 (s, 2H), 1.47 (s, 18H), 1.35 (t, *J* = 7.1 Hz, 3H), 1.25 (t, *J* = 7.1 Hz, 3H). ^13^C{^1^H} NMR (151 MHz, CDCl_3_) δ 169.5, 164.9, 161.0, 160.2, 144.5, 133.5, 83.8, 61.1, 60.8, 59.4, 38.5, 27.7, 14.2, 14.1. IR (neat): υ_max_ 1733, 1369, 1141, 1109, 1023, 840, 734 cm^–1^. HRMS (ESI) Calcd for C_21_H_32_O_9_N (M + H)^+^: 442.2071. Found 442.2075.

### 4-Ethoxy-2-(4-(ethoxycarbonyl)oxazol-5-yl)-4-oxobutanoic Acid (**7**)

To a stirred solution of **6** (150 mg, 0.34 mmol) in CH_2_Cl_2_ (0.6 mL) was added CF_3_COOH (0.6 mL). After stirring for 30 min at room temperature, the reaction mixture was evaporated under reduced pressure at room temperature. The crude residue was recrystallized from Et_2_O/hexanes to afford the monoacid **7** (56 mg, 58%) as a white solid. ^1^H NMR (600 MHz, CDCl_3_) δ 8.21 (d, *J* = 1.5 Hz, 1H), 4.47 (t, *J* = 7.1 Hz, 1H), 4.41–4.30 (m, 2H), 4.19–4.11 (m, 2H), 3.23 (ddd, *J* = 17.4, 7.4, 1.9 Hz, 1H), 3.13 (ddd, *J* = 17.4, 6.9, 2.3 Hz, 1H), 1.36 (td, *J* = 7.2, 1.7 Hz, 3H), 1.24 (td, *J* = 7.1, 1.7 Hz, 3H). ^13^C{^1^H} NMR (151 MHz, CDCl3) δ 170.4, 160.8, 144.4, 133.7, 61.5, 61.4, 41.0, 34.0, 14.3, 14.1. IR (neat): υ_max_ 1727, 1317, 1177, 1110, 1022 cm^–1^. Melting point: 76–78 °C. HRMS (ESI) Calcd for C_12_H_16_O_7_N (M + H)^+^: 286.0921. Found 286.0919.

### 4-Benzyl 1-(*tert*-Butyl) 2-(((Benzyloxy)carbonyl)glycyl)succinate (**11**)

To a stirred solution of **10** (1.0 g, 3.2 mmol, 1 equiv) in tetrahydrofuran (10 mL) was added to a suspension of sodium hydride (60% dispersion in mineral oil, 117 mg, 4.8 mmol, 1.5 equiv, prewashed with hexanes) in tetrahydrofuran (10 mL) maintained at 0 °C under a nitrogen atmosphere. After 20 min, a solution of benzyl bromoacetate (0.62 mL, 3.9 mmol, 1.2 equiv) in tetrahydrofuran (5 mL) was added dropwise, and the mixture was stirred overnight at room temperature. The resulting mixture was quenched with saturated aqueous NH_4_Cl solution and extracted with EtOAc (2 × 15 mL). The combined organic extracts were washed with saturated brine, dried over MgSO_4_, and evaporated under reduced pressure. The residue was chromatographed on silica, eluting with 30% EtOAc in hexanes (*R*_f_ = 0.4) to give the title compound **11** (963 mg, 65%) as a pale-yellow oil. ^1^H NMR (600 MHz, CDCl_3_) δ 7.41–7.31 (m, 10H), 5.43 (t, *J* = 5.1 Hz, 1H), 5.15 (s, 2H), 5.14–5.06 (m, 2H), 4.51 (dd, *J* = 19.6, 5.5 Hz, 1H), 4.24 (dd, *J* = 19.6, 4.6 Hz, 1H), 3.96 (dd, *J* = 9.0, 5.5 Hz, 1H), 3.08 (dd, *J* = 17.8, 9.0 Hz, 1H), 2.94 (dd, *J* = 17.8, 5.5 Hz, 1H), 1.46 (s, 8H), 1.42 (s, 1H).^13^C{^1^H} NMR (151 MHz, CDCl_3_) δ 200.2, 171.1, 166.3, 156.0, 136.2, 135.4, 128.6, 128.5, 128.4, 128.3, 128.2, 128.1, 83.5, 67.0, 66.9, 52.2, 51.1, 32.4, 27.8. IR (neat): υ_max_ 3379, 1714, 1455, 1246, 1146, 697 cm^–1^. HRMS (ESI) Calcd for C_25_H_29_O_7_N^2^^3^Na (M + Na)^+^: 478.1836. Found 478.1844.

### 4-Benzyl 1-(*tert*-Butyl) 2-(((Benzyloxy)carbonyl)glycyl)-2-fluorosuccinate (**12**)

To a suspension of NaH (60% dispersion in mineral oil, 75.6 mg, 3.15 mmol, 1.5 equiv, prewashed with hexanes) in anhydrous THF (5 mL) was added β-keto ester **11** (0.96 g, 2.1 mmol) in THF (10 mL) dropwise at 0 °C under argon. After 30 min of stirring at this temperature, the reaction mixture was warmed to room temperature and stirred for 2 h. Then, a solution of Selectfluor (0.82 g, 2.31 mmol, 1.1 equiv) in DMF (5 mL) was added through a syringe. After stirring for 12 h at room temperature, the reaction was quenched with water and extracted with ethyl acetate (2 × 15 mL). The residue was subjected to silica gel flash chromatography, eluting with 40% EtOAc in hexanes (*R*_f_ = 0.55) to give the title compound **12** (758 mg, 76%) as a colorless oil. ^1^H NMR (400 MHz, CDCl_3_) δ 7.41–7.29 (m, 10H), 5.22 (t, *J* = 5.3 Hz, 1H), 5.16–5.04 (m, 4H), 4.47 (dd, *J* = 5.2, 2.6 Hz, 2H), 3.46 (dd, *J* = 33.2, 17.7 Hz, 1H), 3.20 (dd, *J* = 17.7, 14.9 Hz, 1H), 1.46 (s, 8H), 1.40 (s, 1H). ^13^C{^1^H} NMR (101 MHz, CDCl_3_) δ 200.31 (d, *J* = 28.7 Hz), 167.9, 162.9 (d, *J* = 23.5 Hz), 156.0, 135.6 (d, *J* = 140.2 Hz), 128.9 (d, *J* = 18.3 Hz), 128.7, 128.6, 128.5, 128.4, 128.2, 128.1, 97.5 (d, *J* = 199.8 Hz), 85.4, 67.3, 67.0, 47.9 (d, *J* = 5.1 Hz), 39.4 (d, J = 21.6 Hz), 27.6. ^19^F NMR (376 MHz, CDCl3) δ −170.3 (dd, *J* = 33.2, 14.9 Hz). IR (neat): υ_max_ 3409, 1732, 1519, 1249, 1154, 697 cm^–1^. HRMS (ESI) Calcd for C_25_H_28_O_7_NF^23^Na (M + Na)^+^: 496.1742. Found 496.1742.

### 3-(4-(Ethoxycarbonyl)oxazol-5-yl)propanoic Scid (**20**)

Ethyl isocyanoacetate (200 mg, 1.76 mmol, 1 equiv) in dry dimethylformamide (4 mL) was treated with a solution of 1,8-diazabicyclo[5.4.0]undec-7- ene (396 μL, 2.65 mmol, 1.5 equiv) followed by a solution of the succinic anhydride (228 mg, 2.2 mmol, 1.3 equiv) in DMF (2 mL) and the mixture was heated at 80 °C in an oil bath for 6 h. It was poured into water, extracted with ethyl acetate (3 × 5 mL), the extract was washed with water, dried over Na_2_SO_4_ and evaporated. The desired compound **20** obtained as a colorless liquid (271 mg, 72%) was carried forward without further purification. ^1^H NMR (800 MHz, CDCl_3_) δ 7.76 (s, 1H), 4.32 (q, *J* = 7.2 Hz, 2H), 3.32 (t, *J* = 7.5 Hz, 2H), 2.73 (t, *J* = 7.6 Hz, 2H), 1.33 (t, *J* = 7.2 Hz, 3H). ^13^C{^1^H} NMR (201 MHz, CDCl_3_) δ 176.9, 161.7, 157.7, 149.4, 127.4, 61.2, 31.3, 21.2, 14.2. HRMS (ESI) Calcd for C_9_H_12_O_5_N (M + H)^+^: 214.0710. Found 214.0709.

### 5-Amino-4-oxopentanoic Acid Hydrochloride (**21**)

To a stirred solution of **20** (271 mg, 1.27 mmol, 1 equiv) in THF (1 mL) was added 5 mL of 6 N hydrochloric acid and stirred at 100 °C. After 4 h the solvent was removed on a rotary evaporator to obtain a tan solid. The crude material, upon crystallization from ethanol/Et_2_O (TLC: ^*n*^BuOH:H_2_O:CH_3_CO_2_H, 12:5:3, *R*_f_ = 0.3), provided the desired compound **21** (189 mg, 89%) as colorless needles, respectively. The spectral details are consistent with the reported literature.^30^

### (2*R*,3*R*)-2-(Azidomethyl)-3-fluoro-5-methoxytetrahydrofuran (**26**)

The alcohol to trifluoromethanesulfonate conversion procedure was adapted from our prior work.^31^ To a solution of **25** (200 mg, 1.16 mmol, 1 equiv) and pyridine (205 μL, 2.54 mmol, 2.2 equiv) in CH_2_Cl_2_ (2 mL) was cooled to 0 °C. A separate vial containing trifluoromethanesulfonic anhydride (390 μL, 2.32 mmol, 2.0 equiv) in CH_2_Cl_2_ (2 mL) was cooled to 0 °C, and this mixture was added dropwise to solution of **25** with vigorous stirring. The mixture was stirred at 0 °C for 15 min, then diluted with hexanes (4 mL). A white powder precipitated and was filtered away, and the supernatant was concentrated at 0 °C to give 220 mg of crude **26**, colorless oil and was used directly without further purification.

### (2*R*,3*R*)-2-(Azidomethyl)-3-fluoro-5-methoxytetrahydrofuran (**27**)

To a solution of crude triflate **26** (220 mg) in THF (4 mL) was treated with tetra-*n*-butylammonium fluoride in tetrahydrofuran (1 M, 2.9 mL, 2.9 mmol) at 0 °C. The reaction mixture was stirred at room temperature for 2 h under a stream of N_2_. The mixture was diluted with water (10 mL) and EtOAc (10 mL) and the phases were separated. The aqueous phase was washed with another portion of EtOAc (5 mL) and the organics were collected and dried over Na_2_SO_4_, filtered, and concentrated. The residue was chromatographed on silica, eluting with 10% EtOAc in hexanes (*R*_f_ = 0.6) to give the title compound **27** (20.2 mg, 10% yield over two steps) as a colorless oil. ^1^H NMR (600 MHz, CDCl_3_) δ 5.25 (dt, *J* = 5.8, 3.0 Hz, 1.5H), 5.16 (ddd, *J* = 5.8, 3.2, 1.4 Hz, 0.5H), 4.16 (dddd, *J* = 26.7, 7.2, 5.5, 3.2 Hz, 1H), 3.61 (ddd, *J* = 12.9, 7.3, 1.6 Hz, 1H), 3.51 (dd, *J* = 12.9, 5.5 Hz, 1H), 3.40 (s, 3H), 2.48 (dddd, *J* = 30.6, 15.4, 5.8, 1.4 Hz, 1H), 2.26 (dddd, *J* = 30.4, 15.5, 5.8, 2.8 Hz, 1H). ^13^C{^1^H} NMR (151 MHz, CDCl_3_) δ 104.2, 92.8 (d, *J* = 183.2 Hz), 78.7 (d, *J* = 19.3 Hz), 55.5, 49.2 (d, *J* = 11.3 Hz), 40.8 (d, *J* = 22.3 Hz). ^19^F NMR (565 MHz, CDCl_3_) δ −192.03 (dtd, *J* = 56.9, 30.6, 26.7 Hz). IR (neat): υ_max_ 2098, 1270, 1178, 1088, 984 cm^–1^. HRMS (ESI) Calcd for C_6_H_10_O_2_N_3_FNa (M + Na)^+^: 198.0649. Found 198.0651.

### (2*R*,3*R*)-2-((1,3-Dioxoisoindolin-2-yl)methyl)-5-methoxytetrahydrofuran-3-yl 4-Methylbenzenesulfonate (**30b**)

To a solution of **29** (200 mg, 0.72 mmol) in CH_2_Cl_2_ (8 mL) at 0 °C, was added DMAP (107 mg, 0.86 mmol, 1.2 equiv) followed by TsCl (163 mg, 0.86 mmol, 1.2 equiv) and the reaction was allowed to reach room temperature and stirring was continued for 12 h. After the completion of reaction, the mixture was concentrated on vacuum and chromatographed on silica, eluting with 30% EtOAc in hexanes (*R*_f_ = 0.5) to give the title compound (186 mg, 60%) as a white solid. ^1^H NMR (600 MHz, CDCl_3_) δ 7.89 (dd, *J* = 5.4, 3.0 Hz, 2H), 7.76 (dd, *J* = 5.5, 3.0 Hz, 2H), 7.73–7.66 (m, 2H), 7.32–7.25 (m, 2H), 5.05 (dd, *J* = 5.2, 0.8 Hz, 1H), 4.94 (ddd, *J* = 7.7, 2.9, 1.7 Hz, 1H), 4.54 (ddd, *J* = 8.3, 5.6, 2.9 Hz, 1H), 3.82 (dd, *J* = 14.0, 5.7 Hz, 1H), 3.72 (dd, *J* = 14.1, 8.0 Hz, 1H), 3.34 (s, 3H), 2.42 (s, 3H), 2.24 (ddd, *J* = 14.9, 7.7, 5.2 Hz, 1H), 2.05 (ddd, *J* = 14.9, 1.7, 0.9 Hz, 1H).^13^C{^1^H} NMR (151 MHz, CDCl_3_) δ 168.2, 144.8, 134.1, 133.5, 132.0, 129.8, 127.9, 123.4, 104.5, 81.0, 80.3, 55.1, 39.2, 38.6, 21.7. Melting point: 120–122 °C (decomposes). HRMS (ESI) Calcd for C_21_H_22_O_7_N^3^^2^S (M + H)^+^: 432.1111. Found 432.1116. [α]_D_^20^ + 680.77 (*c* 1.10, CHCl_3_).

### 4-Benzyl 1-Methyl (*R*)-2-Fluorosuccinate (**34**)

To a stirred solution of diethyaminosulfur trifluoride (0.277 mL, 2.1 mmol, 1 equiv) in CH_2_Cl_2_ (2 mL) cooled to 0 °C was added dropwise to a solution of **33** (500 mg, 2.1 mmol, 1 equiv) in CH_2_Cl_2_ (5 mL). The mixture was allowed to reach ambient temperature for 1 h and water (5 mL) was added cautiously to the vigorously stirred solution. The organic layer was separated, washed with sat. NaHCO_3_ and sat. NaCl, dried (MgSO_4_), and evaporated under reduced pressure. The residue was chromatographed on silica, eluting with 30% EtOAc in hexanes (*R*_f_ = 0.5) to give the title compound **34** (348 mg, 69%) as a colorless oil. ^1^H NMR (600 MHz, CDCl_3_) δ 7.43–7.33 (m, 5H), 5.34 (ddd, *J* = 47.2, 6.6, 4.8 Hz, 1H), 5.20 (dd, *J* = 19.1, 12.2 Hz, 2H), 3.04 (dd, *J* = 24.6, 4.7 Hz, 1H), 3.03 (dd, *J* = 23.5, 6.7 Hz, 1H). ^13^C{^1^H} NMR (151 MHz, CDCl_3_) δ 169.0 (d, *J* = 22.8 Hz), 168.5 (d, *J* = 3.2 Hz), 135.2, 128.6, 128.5, 128.4, 85.1 (d, *J* = 187.4 Hz), 67.2, 52.7, 37.4 (d, *J* = 23.0 Hz). ^19^F NMR (376 MHz, CDCl_3_) δ −191.11 (dd, *J* = 47.6, 23.9 Hz). IR (neat): υ_max_ 3379, 1714, 1455, 1246, 1146, 697 cm^–1^. HRMS (ESI) Calcd for C_12_H_13_O_4_F^2^^3^Na (M + Na)^+^: 263.0690. Found 263.0691. [α]_D_^20^ + 17.82 (*c* 1.10, CHCl_3_).

### Benzyl (*S*)-3-Hydroxy-4-oxo-4-(*p*-tolylthio)butanoate (**36**)

To a stirred solution of hydroxy acid **32** (3 g, 13.3 mmol, 1 equiv) in dry ethyl acetate (2 mL/mmol) was added 4-methylbenzenethiol (1.74 g, 14 mmol, 1.05 equiv) and 1-hydroxybenzotriazole (2.37 g, 19.9 mmol, 1.5 equiv) at 0 °C followed by *N*-dicyclohexylcarbodiimide (2.73 g, 13.3 mmol, 1 equiv). The mixture was stirred for 24 h at room temperature. At the end of the reaction, a few drops of 50% acetic acid in ethyl acetate were added. The mixture was filtered through Celite, and the organic phase was washed with NaHCO_3_ solution and brine, dried over MgSO_4_, filtered, and concentrated in vacuo. The residue was chromatographed on silica, eluting with 20% EtOAc in hexanes (*R*_f_ = 0.4) to give the title compound **36** (3.3 g, 75%) as a yellow solid. ^1^H NMR (600 MHz, CDCl_3_) δ 7.43–7.33 (m, 5H), 7.29 (d, *J* = 6.6 Hz, 2H), 7.25 (d, *J* = 8.0 Hz, 2H), 5.23–5.14 (m, 2H), 4.67 (dd, *J* = 6.6, 4.2 Hz, 1H), 3.06–2.91 (m, 2H), 2.40 (s, 3H). ^13^C{^1^H} NMR (151 MHz, CDCl_3_) δ 201.3, 171.2, 139.9, 135.2, 134.6, 130.1, 128.7, 128.5, 128.4, 123.2, 74.4, 67.1, 38.4, 21.4. IR (neat): υ_max_ 3477, 1734, 1700, 1455, 1236, 1169, 808, 697 cm^–1^. Melting point: 78–80 °C (decomposes). HRMS (ESI) Calcd for C_18_H_18_O_4_^2^^3^Na^3^^2^S (M + Na)^+^: 353.0818. Found 353.0814. [α]_D_^20^ + 53.24 (*c* 1.10, CHCl_3_). 99% ee; Chiral HPLC analysis of the product: Daicel Chiralpak IA 250 × 4.6 mm 5um column; hexane/2-propanol = 90/10, detected at 210 nm, Flow rate = 1 mL/min, Retention times: 14.6 min (S), 18.5 min (R).

### Benzyl (*R*)-3-Fluoro-4-oxo-4-(*p*-tolylthio)butanoate (**35**) from 4-Benzyl 1-Methyl (*R*)-2-Fluorosuccinate (**34**)

A stirred solution of **34** in THF (1 g, 4.2 mmol, 1 equiv) was refluxed with 5% sulfuric acid (10 mL) for 3 h. The reaction was cooled and extracted with ether (3 × 10 mL). The combined organics were dried over MgSO_4_ and concentrated in vacuo. The desired acid intermediate obtained as a colorless liquid (706 mg, 75%) was carried forward without further purification. ^1^H NMR (600 MHz, CDCl_3_) δ 7.74–7.60 (m, 5H), 5.64 (ddd, J = 47.1, 6.5, 4.6 Hz, 1H), 5.53–5.43 (m, 2H), 3.38 (dd, J = 5.5, 4.4 Hz, 1H), 3.34 (dd, J = 5.5, 3.9 Hz, 1H). To a stirred solution of crude acid (706 mg, 3.1 mmol, 1 equiv) and 1-hydroxybenzotriazole monohydrate (506 mg, 3.7 mmol, 1.2 equiv) in dichloromethane (15 mL) at 0 °C was added 1-ethyl-3-(3-(dimethylamino)propyl)carbodiimide hydrochloride (710 mg, 3.7 mmol, 1.2 equiv) portion wise. After stirring for 30 min, 4-methylbenzenethiol (458 mg, 3.7 mmol, 1.2 equiv) was added to the solution, and the mixture was allowed to warm to room temperature and stirred for 12 h. The reaction was quenched with saturated aqueous NaHCO_3_ solution and extracted with ethyl acetate (2 × 10 mL). The combined organic layer was washed with brine, dried over Na_2_SO_4_, and concentrated. The residue was chromatographed on silica, eluting with 20% EtOAc in hexanes (*R*_f_ = 0.5) to give the title compound **35** (0.85 g, 82%) as a pale-yellow gummy oil.

^1^H NMR (600 MHz, CDCl_3_) δ 7.44–7.36 (m, 5H), 7.32 (dd, *J* = 8.2, 1.8 Hz, 2H), 7.27 (d, *J* = 8.0 Hz, 2H), 5.48 (dddt, *J* = 47.8, 7.9, 3.9, 0.9 Hz, 1H), 5.23 (s, 2H), 3.18–2.95 (m, 2H), 2.42 (s, 3H). ^13^C{^1^H} NMR (151 MHz, CDCl_3_) δ 197.5 (d, *J* = 28.0 Hz), 168.4, 140.3, 135.3, 134.7, 130.3, 128.7, 128.5, 128.4, 121.9 (d, *J* = 5.5 Hz), 92.0 (d, *J* = 190.2 Hz), 67.2, 37.8 (d, *J* = 22.1 Hz), 21.4. ^19^F NMR (565 MHz, CDCl3) δ −187.8 (ddd, J = 49.1, 29.0, 21.0 Hz). IR (neat): υ_max_ 1737, 1697, 1262, 1168, 807, 696 cm^–1^. HRMS (ESI) Calcd for C_18_H_17_O_3_FNaS (M + Na)^+^: 355.0774. Found 355.0771. [α]_D_^20^ + 78.57 (*c* 1.10, CHCl_3_). Assuming >98% ee; Chiral HPLC analysis of the product: Daicel Chiralpak IA 250 × 4.6 mm 5um column; hexane/2-propanol = 90/10, detected at 210 nm, Flow rate = 1 mL/min, Retention times: 7.7 min (S).

### Benzyl (*R*)-3-Fluoro-4-oxo-4-(*p*-tolylthio)butanoate (**35**) from Benzyl (*S*)-3-Hydroxy-4-oxo-4-(*p*-tolylthio)butanoate (**36**)

To a stirred solution of diethyiaminosulfur trifluoride (0.57 mL, 4.36 mmol, 1.2 equiv) in ethanol-free dry chloroform (15 mL) cooled to 0 °C was added dropwise to a solution of **36** (1.2 g, 3.63 mmol, 1 equiv) in chloroform (20 mL). The mixture was stirred for 1 h, and an additional solution of diethyiaminosulfur trifluoride (0.5 equiv) in ethanol-free dry chloroform (5 mL) was added at 0 °C. After 30 min, water (1 mL) was added cautiously to the vigorously stirred solution. The organic layer was separated, washed with saturated sodium bicarbonate solution, and saturated brine, dried over MgSO_4_, and evaporated under reduced pressure. The residue was chromatographed on silica, eluting with 20% EtOAc in hexanes (*R*_f_ = 0.5) to give the title compound **35** (880 mg, 73%) as a pale-yellow gummy oil. The spectral data of compound **35**, obtained by this route, are identical to those of compound **35** synthesized from benzyl (*R*)-3-fluoro-4-oxo-4-(*p*-tolylthio)butanoate (**37**), including optical rotation.

### Benzyl (±)-5-(((Benzyloxy)carbonyl)amino)-3-fluoro-4-oxopentanoate (**13**) from 4-Benzyl 1-(*tert*-Butyl) 2-(((Benzyloxy)carbonyl)glycyl)-2-fluorosuccinate (**12**)

A solution of **12** (500 mg, 1.0 mmol, 1 equiv) and trifluoroacetic acid (0.8 mL, 10.5 mmol, 10.0 equiv) in dichloromethane (10 mL) was heated at reflux overnight. Remove solvent and purification by flash column chromatography on silica, eluting with 40% EtOAc in hexanes (*R*_f_ = 0.4) to give the title compound **13** (335 mg, 85%) as a white solid. The spectral profile of compound **13** is identical to **13a** in all aspects except for its optical rotation.

### Benzyl (*R*)-5-(((Benzyloxy)carbonyl)amino)-3-fluoro-4-oxopentanoate (**13a**) from Benzyl (*R*)-3-Fluoro-4-oxo-4-(*p*-tolylthio)butanoate (**35**)

To a stirred solution of **35** (500 mg, 1.5 mmol, 1 equiv) and benzyl tri-*n*-butylstannylmethylcarbamate **37** (1.37 g, 3.0 mmol, 2 equiv) in dry THF (20 mL) was added copper(I)diphenylphosphinate (0.84 g, 3.0 mmol, 2 equiv) at room temperature under nitrogen. The reaction was heated at 50 °C for 2 h. After cooling, the suspension was filtered through Celite and washed with ethyl acetate. The filtrate was washed with water, and the combined organic layers were washed with brine, dried over Na_2_SO_4_, and concentrated. The residue was chromatographed on silica, eluting with 40% EtOAc in hexanes (*R*_f_ = 0.4) to give the title compound **13a** (511 mg, 91%) as a white solid. [α]_D_^20^ + 21.70 (*c* 1.10, CHCl_3_). 99% ee; Chiral HPLC analysis of the product: Daicel Chiralpak IA 250 × 4.6 mm 5um column; hexane/2-propanol = 75/25, detected at 210 nm, Flow rate = 1 mL/min, Retention times: 13.9 min (S), 15.3 min (R).

^1^H NMR (400 MHz, CDCl_3_) δ 7.43–7.31 (m, 10H), 5.34–5.28 (m, 1H), 5.28–5.19 (m, 1H), 5.18–5.12 (m, 4H), 4.54–4.37 (m, 2H), 3.22–2.98 (m, 2H). ^13^C{^1^H} NMR (151 MHz, CDCl_3_) δ 204.05 (d, *J* = 25.7 Hz), 168.52, 156.17, 135.63 (d, *J* = 190.0 Hz), 135.00, 128.75, 128.70, 128.63, 128.58, 128.56, 128.43, 128.30, 128.26, 128.22, 128.16, 128.12, 91.35 (d, *J* = 186.2 Hz), 67.32, 67.11, 48.34 (d, *J* = 4.4 Hz), 37.18 (d, *J* = 21.6 Hz). ^19^F NMR (376 MHz, CDCl_3_) δ −197.09 – −197.83 (m). IR (neat): υ_max_ 3412, 1716, 1520, 1248, 1167, 738, 697 cm^–1^. Melting point: 58–60 °C (decomposes). HRMS (ESI) Calcd for C_20_H_21_O_5_NF (M + H)^+^: 374.1398. Found 374.1396.

### (±)- or (*R*)-5-Amino-3-fluoro-4-oxopentanoic Acid Hydrochloride (**14** or **14a**) from Benzyl (±)- or (*R*)-5-(((Benzyloxy)carbonyl)amino)-3-fluoro-4-oxopentanoate (**13** or **13a**)

To a stirred solution of **13** or **13a** (300 mg, 0.8 mmol, 1 equiv) in a 4:1 mixture of tetrahydrofuran (12 mL) and 2 M aq. HCl (3 mL) was added 10% palladium on carbon (85 mg, 0.8 mmol, 1 equiv). The reaction vessel was purged with hydrogen and evacuated three times, and the solution was then stirred under a hydrogen atmosphere at 30 °C in an oil bath for 2 h. The suspension was then filtered through Celite, and the residue was washed with a small amount of water. The filtrate was concentrated under reduced pressure to obtain a tan solid. Then, add 2 mL of ethanol to the crude material and heat the mixture to boiling using a sand bath. Once the solution is fully dissolved, slowly add diethyl ether drop by drop until the solution reaches a cloud point and allow the solution to stand until crystals grow. (TLC: ^*n*^BuOH:H_2_O:CH_3_CO_2_H, 12:5:3, *R*_f_ = 0.35), provided the desired compound **14** or **14a** (111 mg, 75% yield) as colorless needles, respectively. ^1^H NMR (600 MHz, DMSO) δ 12.87 (s, 1H), 8.44 (s, 3H), 5.40 (dt, *J* = 46.1, 4.8 Hz, 1H), 4.07 (t, *J* = 14.4 Hz, 2H), 3.01 (t, *J* = 5.4 Hz, 1H), 2.97 (d, *J* = 4.8 Hz, 1H). ^13^C{^1^H} NMR (101 MHz, DMSO) δ 202.3 (d, *J* = 24.1 Hz), 170.9, 91.8 (d, *J* = 181.4 Hz), 45.3, 37.3 (d, *J* = 21.4 Hz). ^19^F NMR (376 MHz, DMSO) δ −193.34 – −198.21 (m). IR (neat): υ_max_ 3022, 1724, 1488, 1397, 1106, 993, 853, 578 cm^–1^. HRMS (ESI) Calcd for C_5_H_9_O_3_NF (M + H)^+^: 150.0561. Found 150.0561. Melting point: 138–140 °C (decomposes). [α]_D_^20^ + 7.02 (*c* 1.10, H_2_O).

Note: A similar route was followed for the synthesis of the other antipode, (*S*)-(−)-5-amino-3-fluoro-4-oxopentanoic acid hydrochloride (**14b**) starting from d-benzyl aspartate. The NMR data of all the compounds in this series were exactly matched with the other enantiomeric series. The optical rotations also showed the same magnitude but with an inverse sign.

## Data Availability

The data underlying this study are available in the published article and its Supporting Information.

## Supporting Information Available

Copies of the ^1^H and ^13^C spectra for all new compounds, X-ray crystallographic data for **14a** and **14b**, and HPLC chromatograms of **36**, **35**, and **13b** (PDF)
