# Supplementary material for: Synthetic Efforts toward the Synthesis of a Fluorinated Analog of 5-Aminolevulinic Acid: Practical Synthesis of Racemic and Enantiomerically Defined 3-Fluoro-5-aminolevulinic Acid
Source: J Org Chem. 2024 Aug 27;89(17):12176–86. doi: 10.1021/acs.joc.4c01070 (PMC11382157; doi:10.1021/acs.joc.4c01070)
Supplement: Supplementary file 1 — jo4c01070_si_001.pdf [file jo4c01070_si_001.pdf]

# Supplementary Information

## Synthetic Efforts toward the Synthesis of a Fluorinated Analog of 5-Aminolevulinic Acid: Practical Synthesis of Racemic and Enantiomerically-defined 3-Fluoro-5-aminolevulinic Acid

Gouthami Pashikanti[‡], Lahu N. Chavan[†ǂ], Lanny S. Liebeskind[‡], and Mark M. Goodman[†ǂ]

[†]Department of Radiology and Imaging Sciences, [††]Department of Neurosurgery, School of Medicine, Emory University, 1364 Clifton Road NE, Atlanta, Georgia 30322

[‡]Department of Chemistry, Emory University, 1515 Dickey Drive, Atlanta, Georgia 30322

[ǂ]Center for Systems Imaging, Emory University, 1841 Clifton Rd NE, Atlanta, Georgia 30322

**Pages**

I. $^{1}$H NMR, $^{13}$C NMR spectra S2 to S19

II. X-ray crystallographic data S20 to S28

III. Copies of HPLC chromatograms S29 to S31

## I. $^{1}$H NMR, $^{13}$C NMR spectra:

## 1,1-Di-*tert*-butyl 2-ethyl 1-(4-(ethoxycarbonyl)oxazol-5-yl)ethane-1,1,2-tricarboxylate (6):

$^{1}$H NMR (600 MHz, $CDCl_3$)

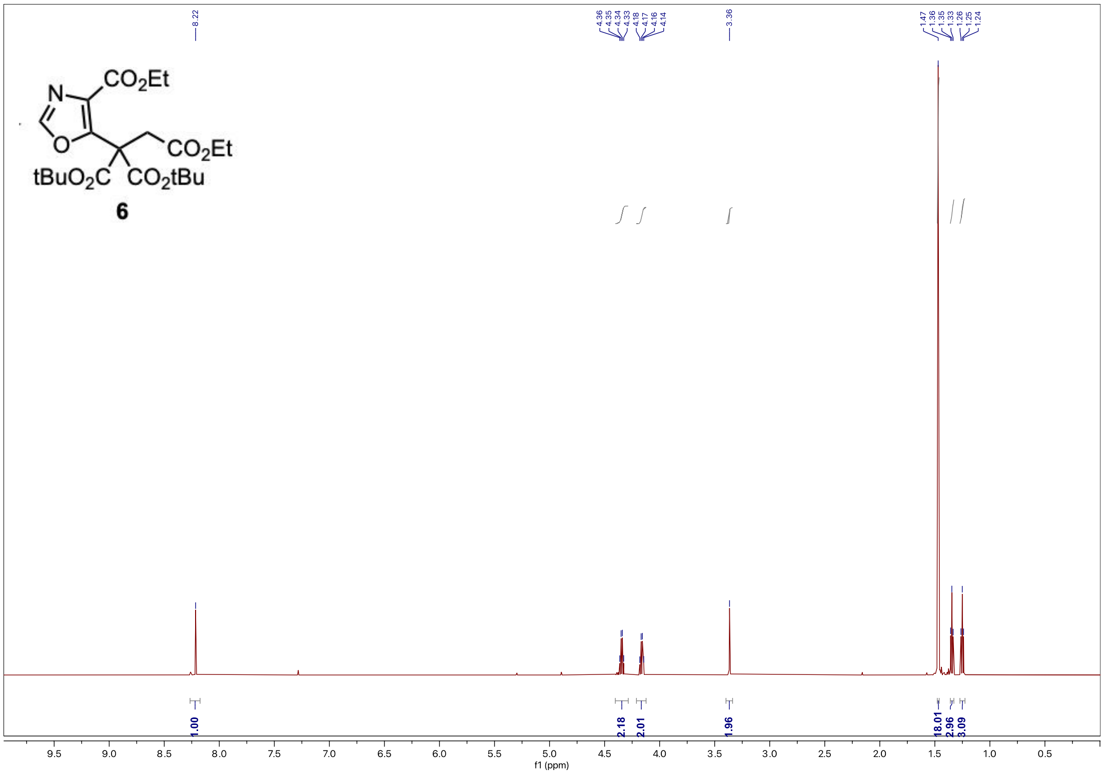


$^{13}$C{1H} NMR (151 MHz, $CDCl_3$)

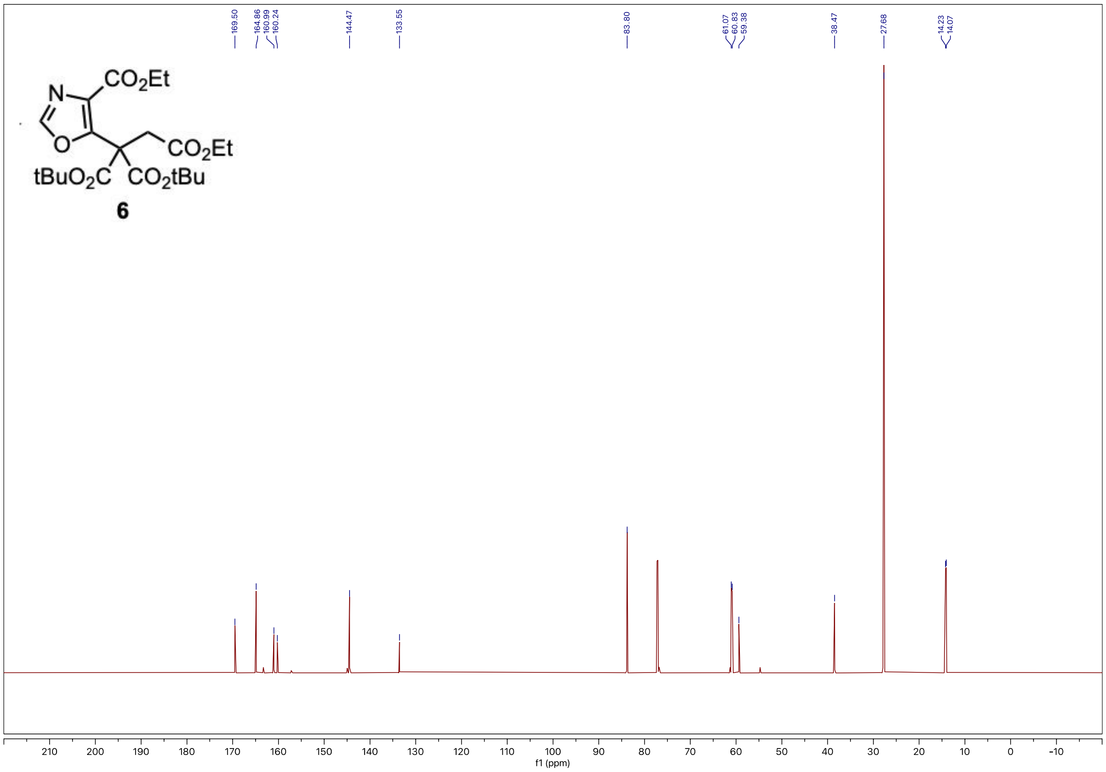

**4-Ethoxy-2-(4-(ethoxycarbonyl)oxazol-5-yl)-4-oxobutanoic acid (7):**

$^{1}H$ NMR (600 MHz, $CDCl_3$)

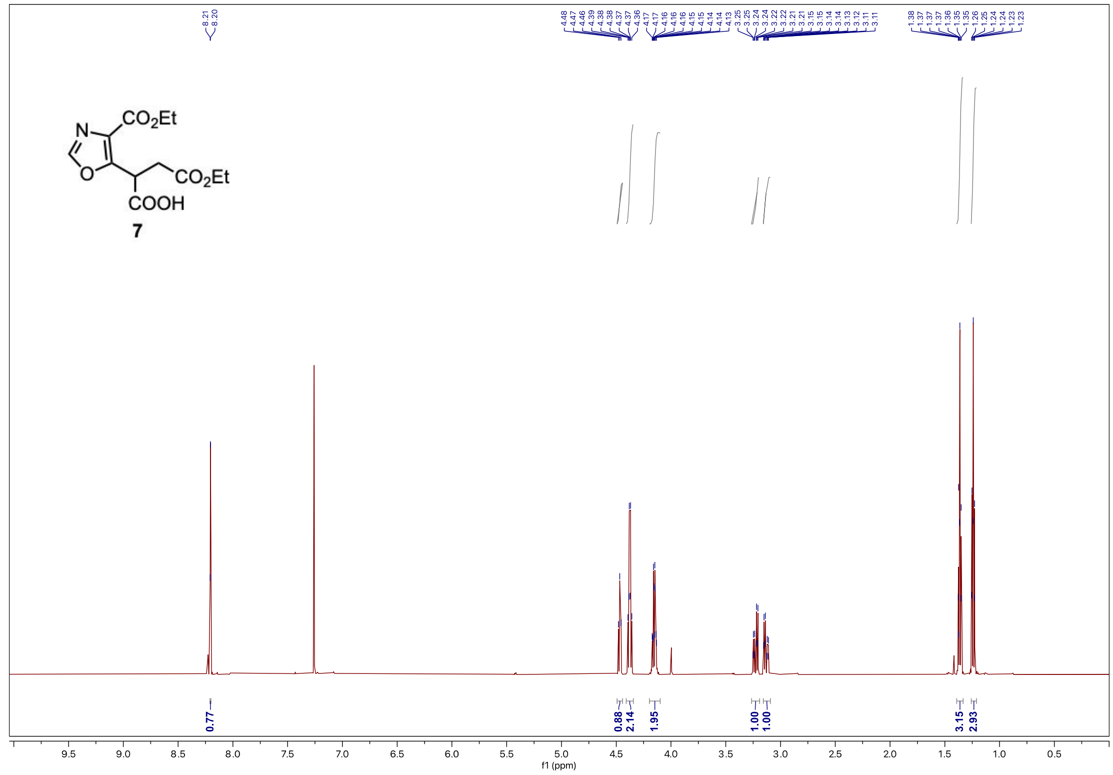


$^{13}C\{1H\}$ NMR (151 MHz, $CDCl_3$)

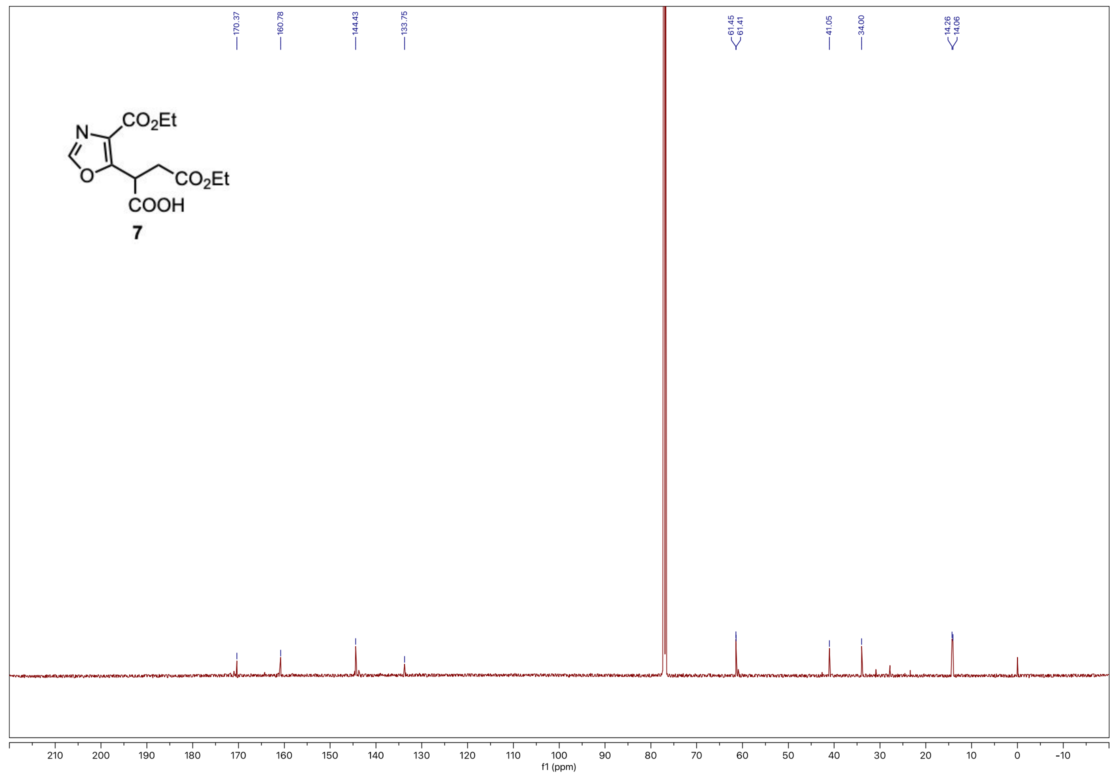

**4-Benzyl 1-(tert-butyl) 2-(((benzyloxy)carbonyl)glycyl)succinate (11):**

$^1$H NMR (600 MHz, $CDCl_3$)

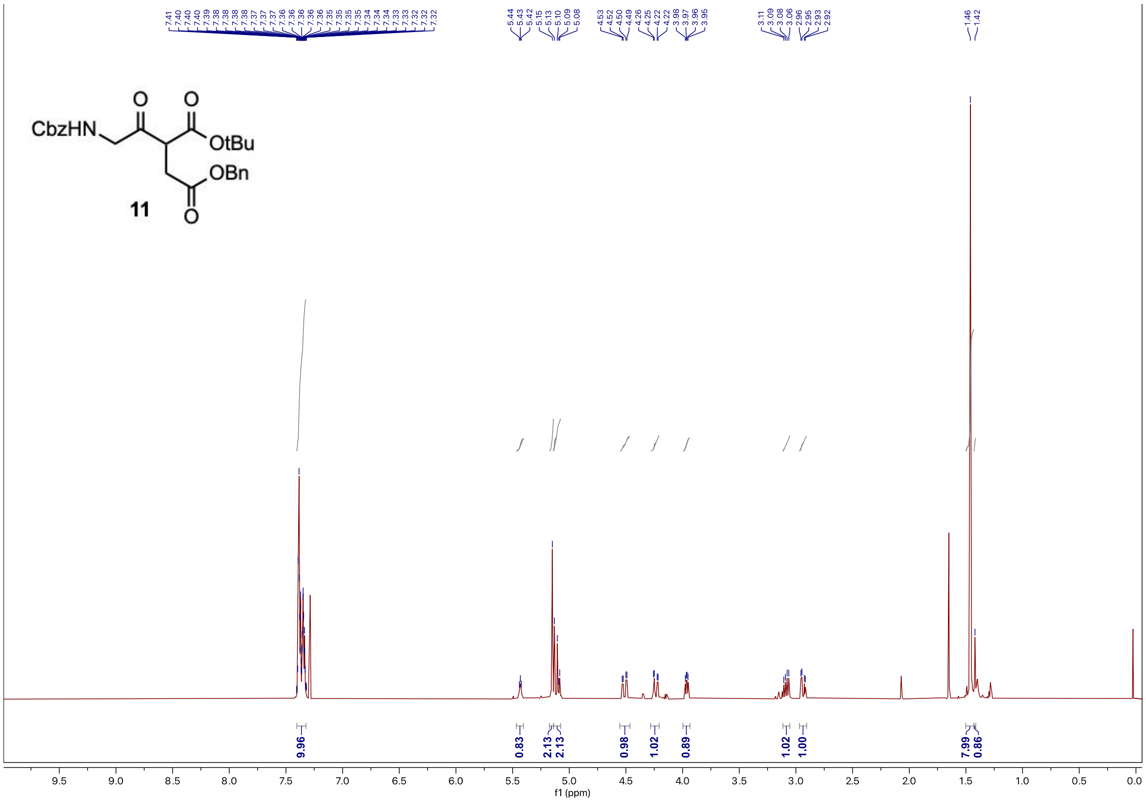

$^{13}$C{1H} NMR (151 MHz, $CDCl_3$)

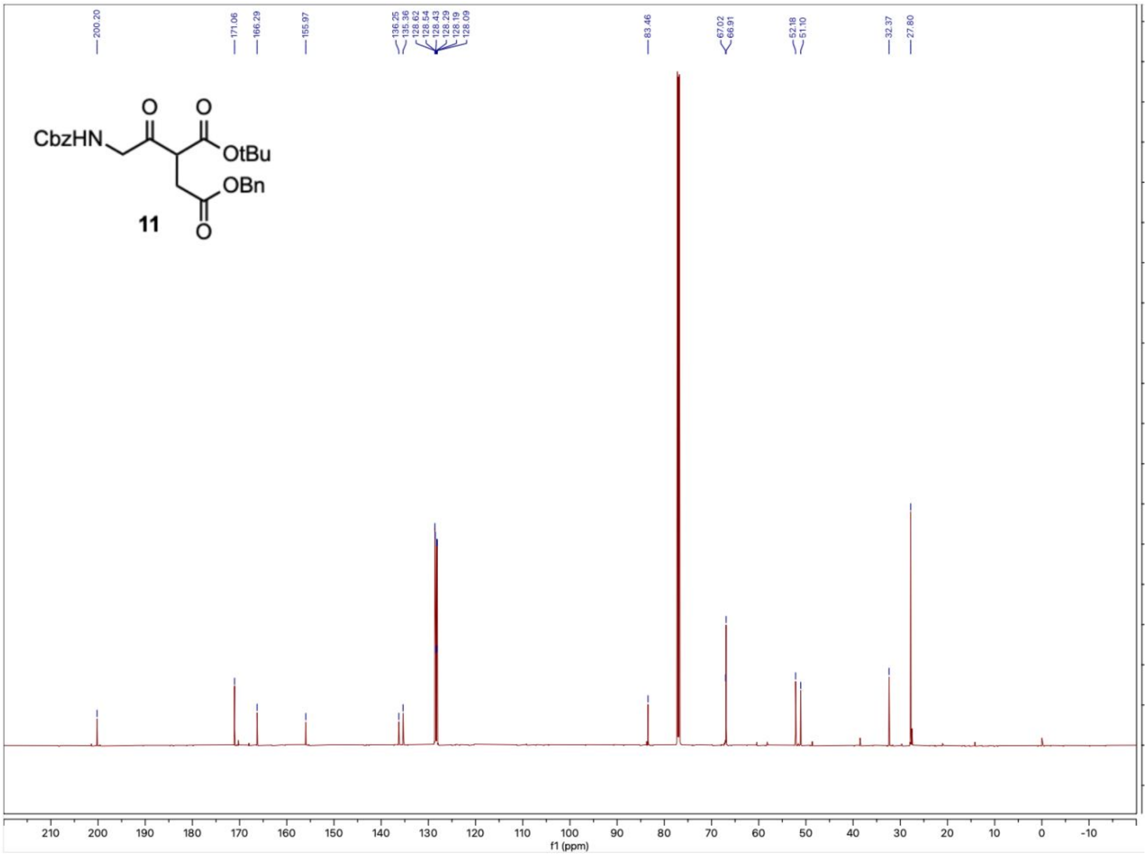

**4-Benzyl 1-(*tert*-butyl) 2-(((benzyloxy)carbonyl)glycyl)-2-fluorosuccinate (12):**

$^{1}$H NMR (400 MHz, $CDCl_3$)

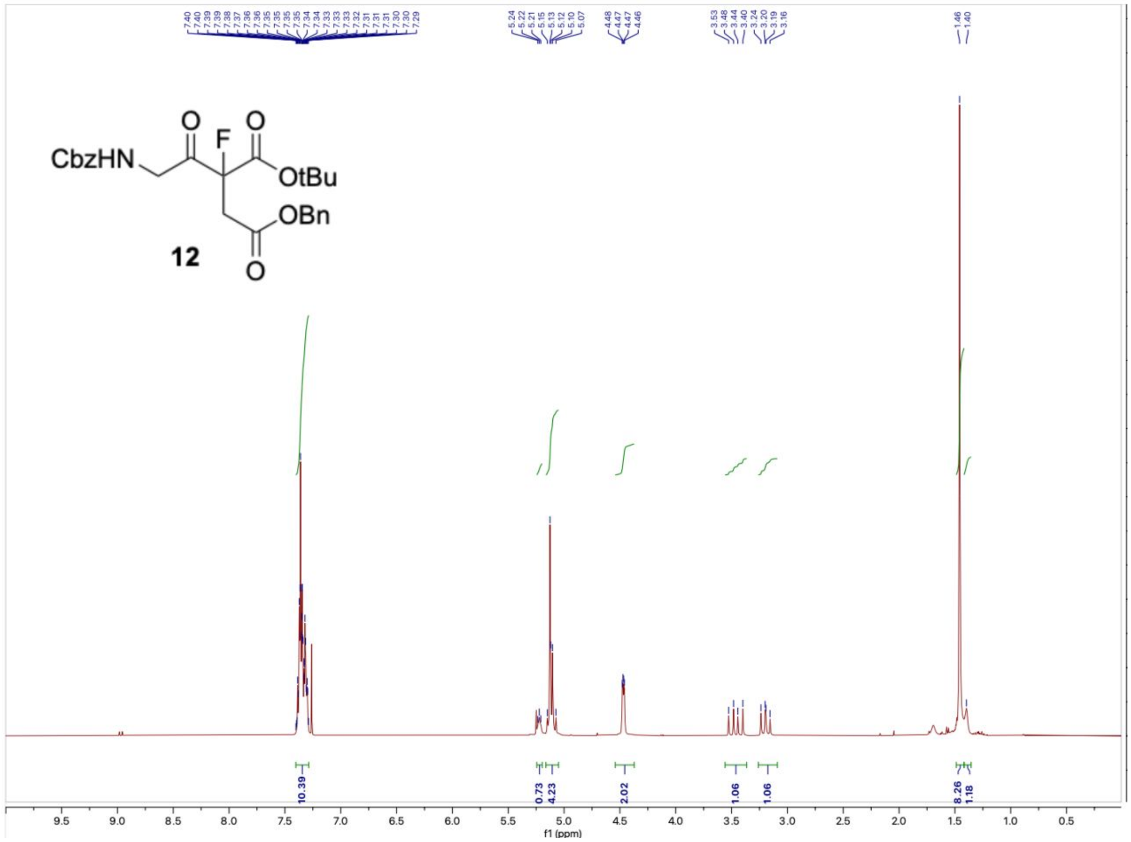


$^{19}$F NMR (376 MHz, CDCl3)

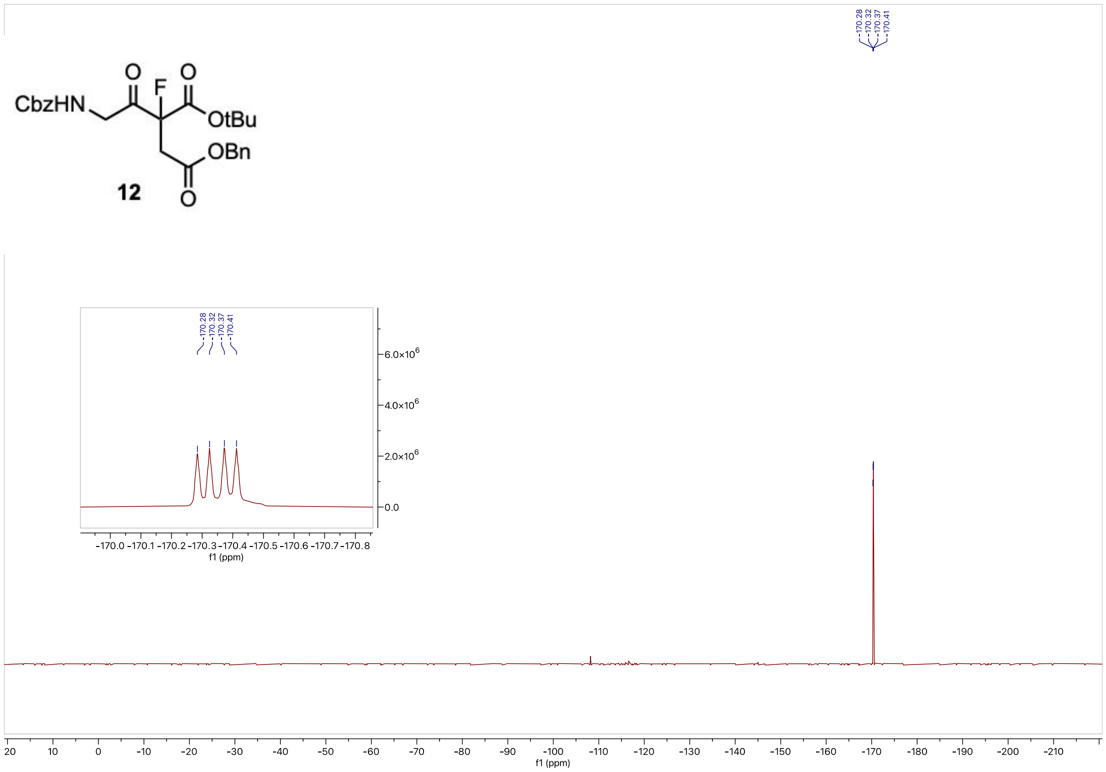

$^{13}C\{1H\}$ NMR (101 MHz, $CDCl_3$)

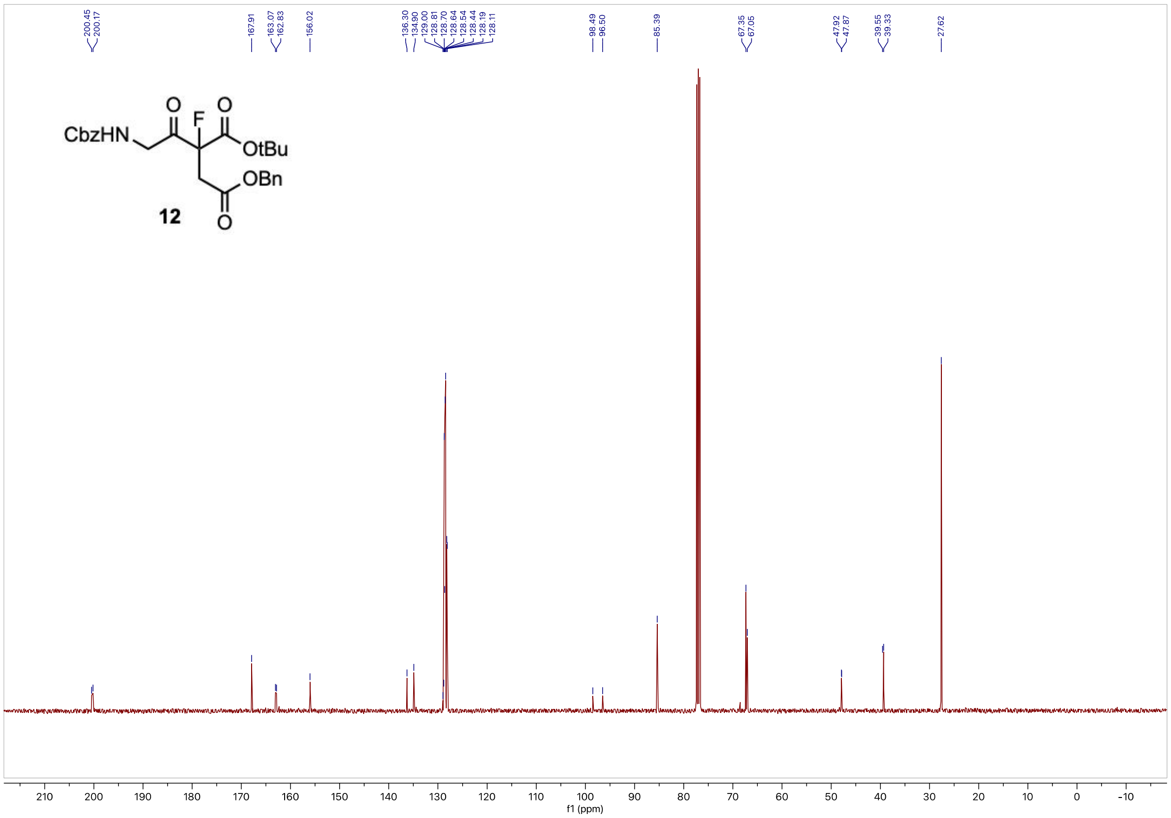

**3-(4-(Ethoxycarbonyl)oxazol-5-yl)propanoic acid (20):**

$^{1}H$ NMR (800 MHz, $CDCl_3$)

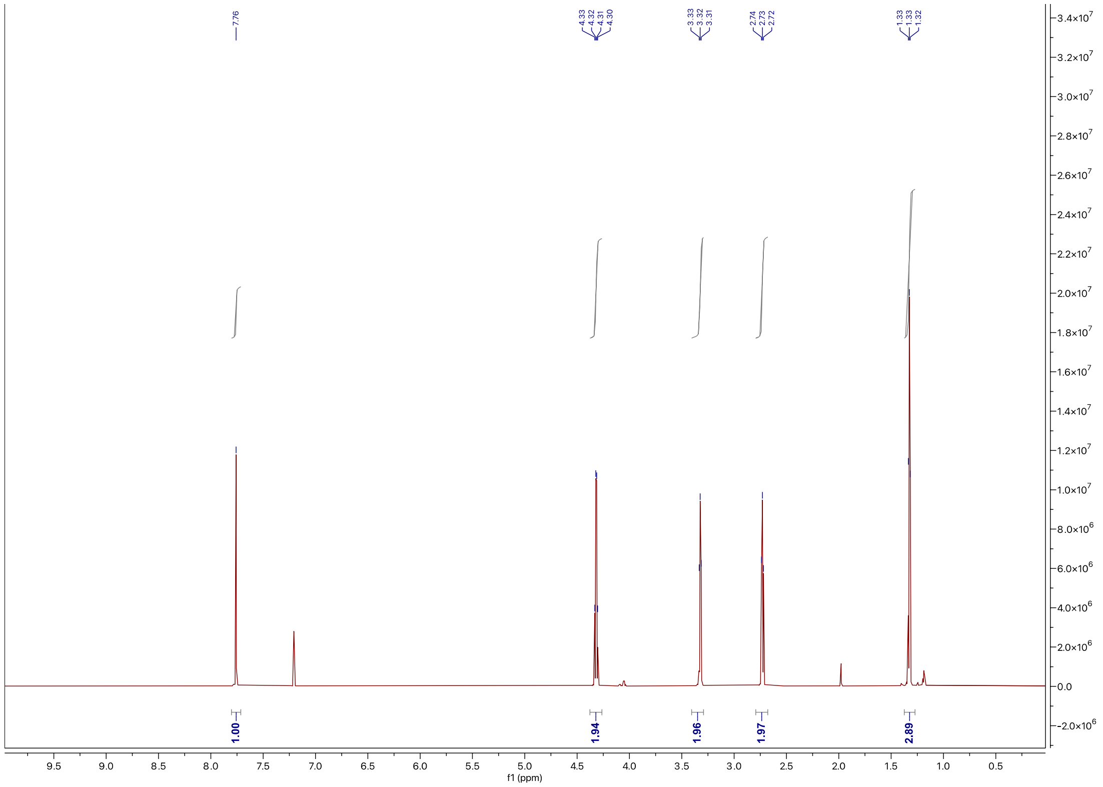


$^{13}C$ {1H} NMR (201 MHz, $CDCl_3$)

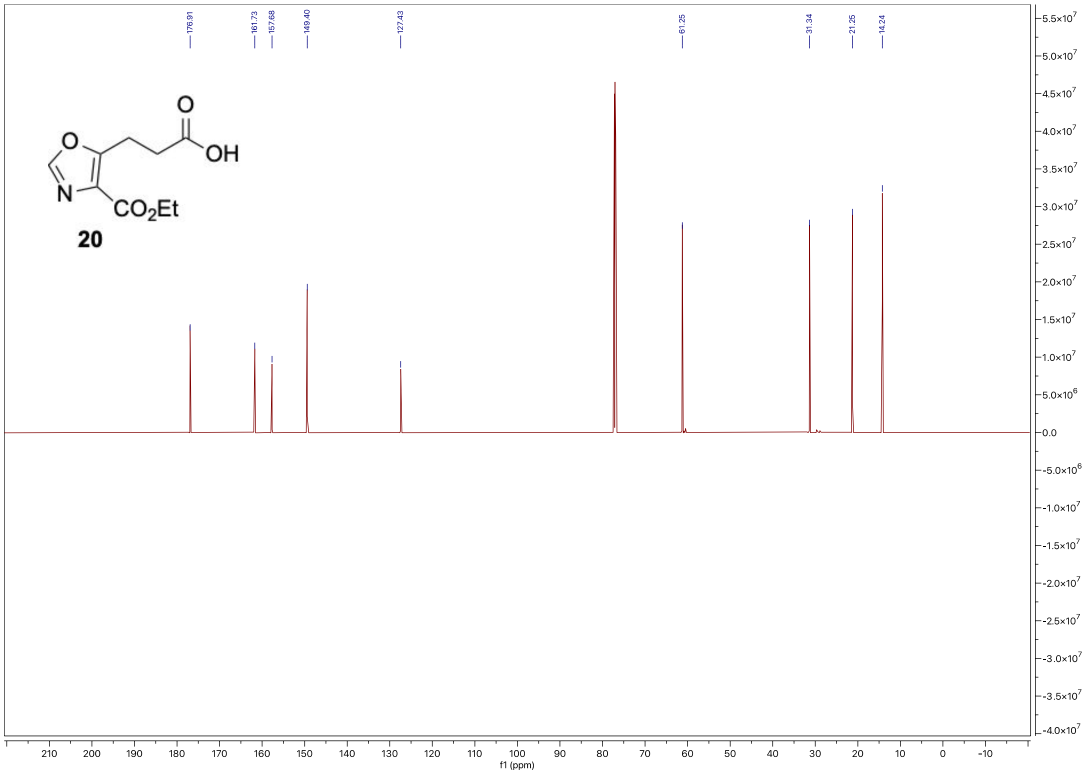

**(2R,3R)-2-(Azidomethyl)-3-fluoro-5-methoxytetrahydrofuran (27):**

$^1$H NMR (600 MHz, $CDCl_3$)

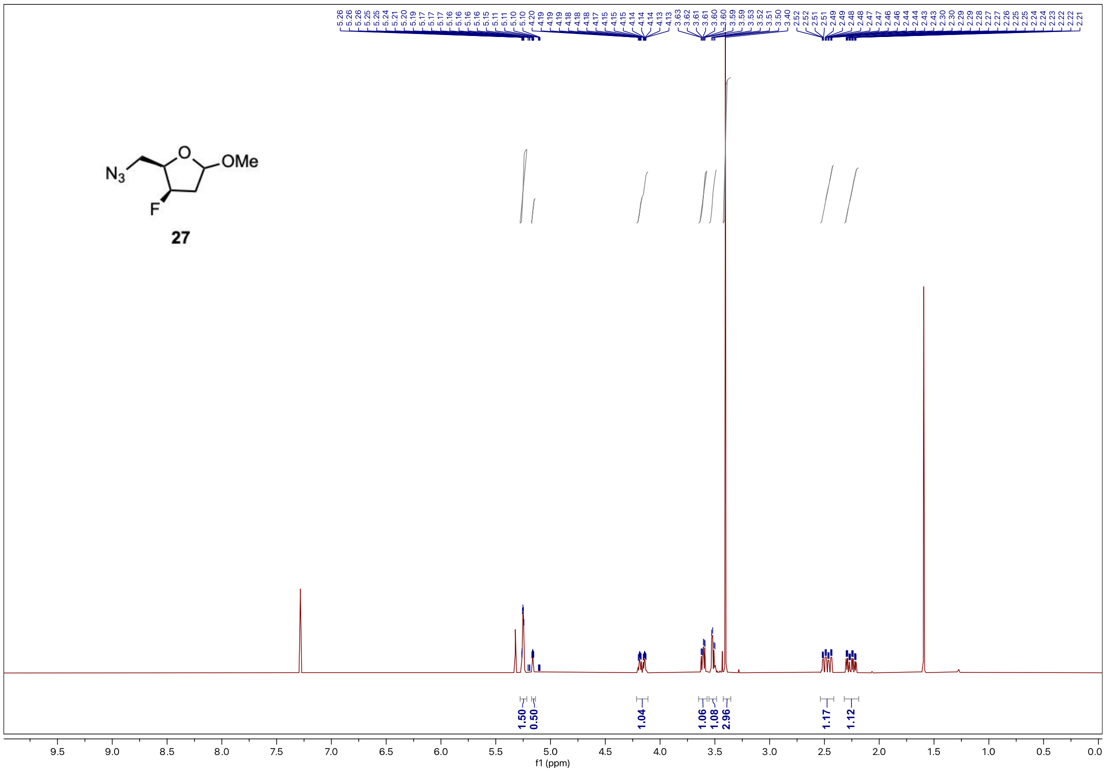


$^{19}$F NMR (565 MHz, $CDCl_3$)

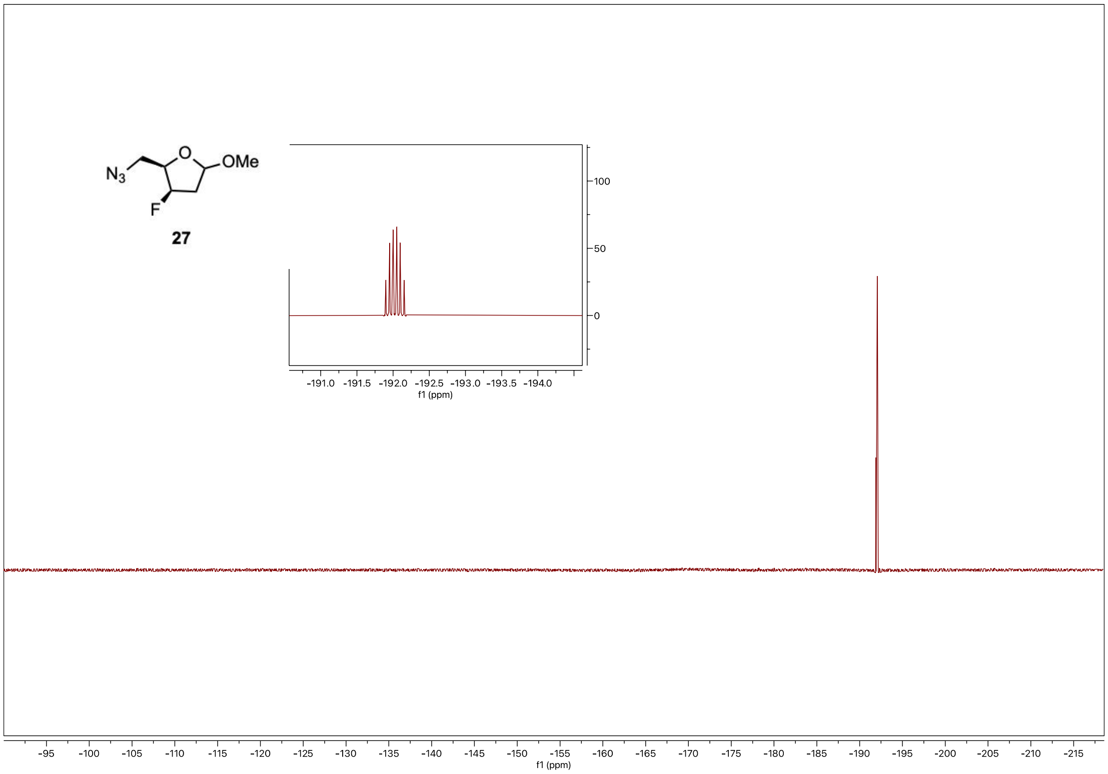

$^{13}C$ {1H} NMR (151 MHz, $CDCl_3$)

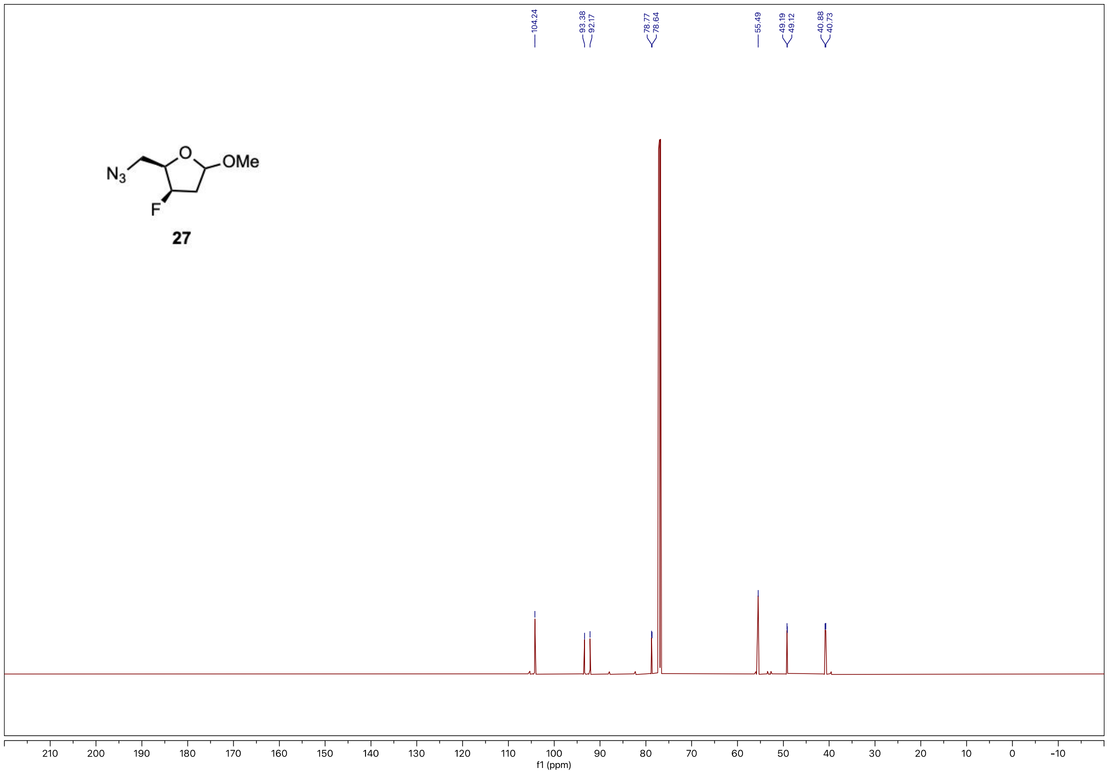

**(2R,3R)-2-((1,3-Dioxoisoindolin-2-yl)methyl)-5-methoxytetrahydrofuran-3-yl 4-methylbenzenesulfonate (30b):**

$^{1}H$ NMR (600 MHz, $CDCl_3$)

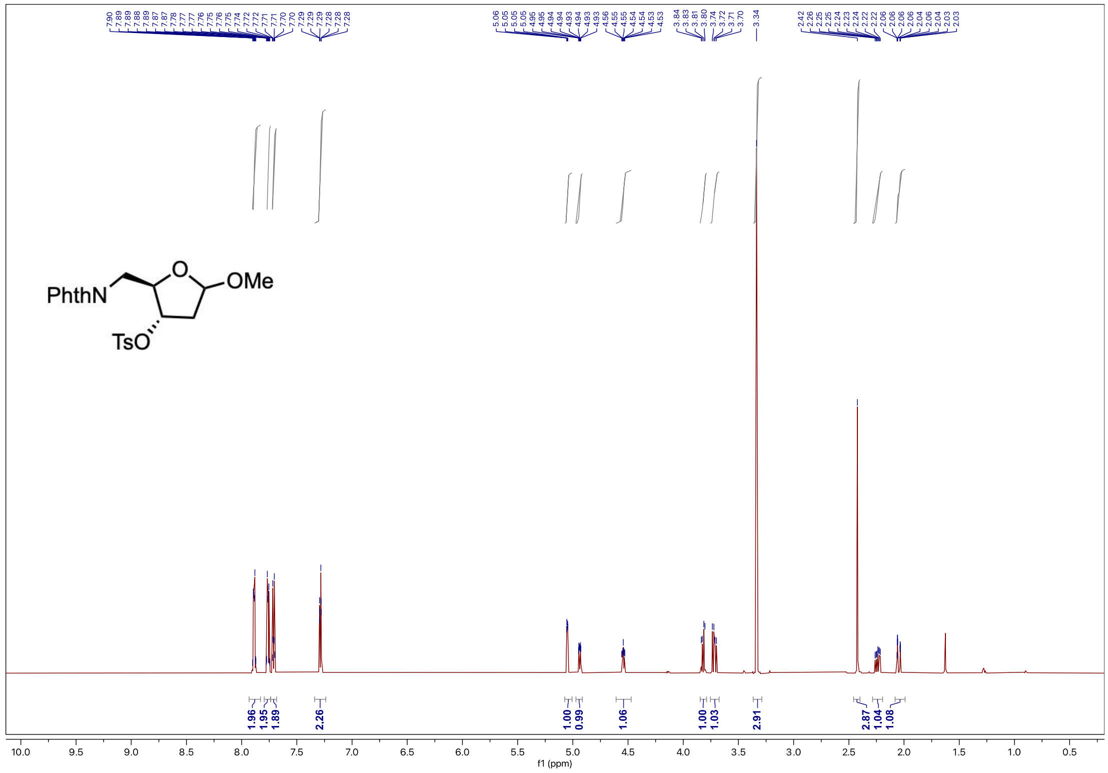

$^{13}C\{1H\}$ NMR (151 MHz, $CDCl_3$)

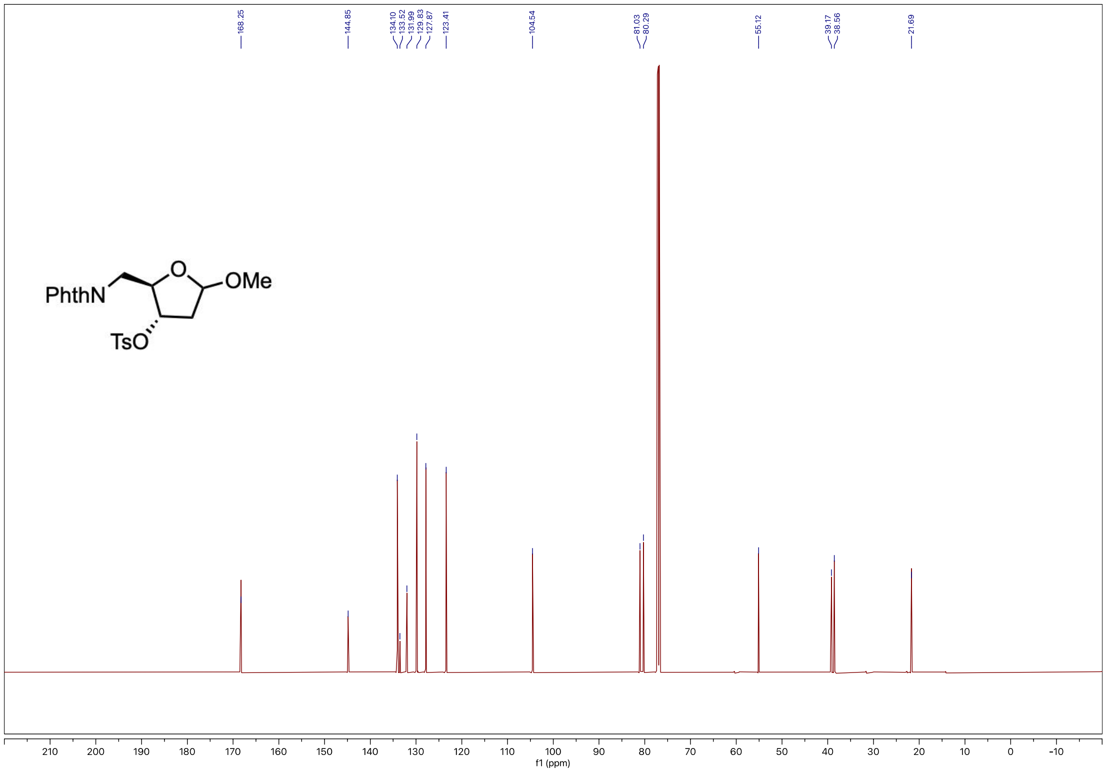

**4-Benzyl 1-methyl (*R*)-2-fluorosuccinate (34):**

$^{1}H$ NMR (600 MHz, $CDCl_3$)

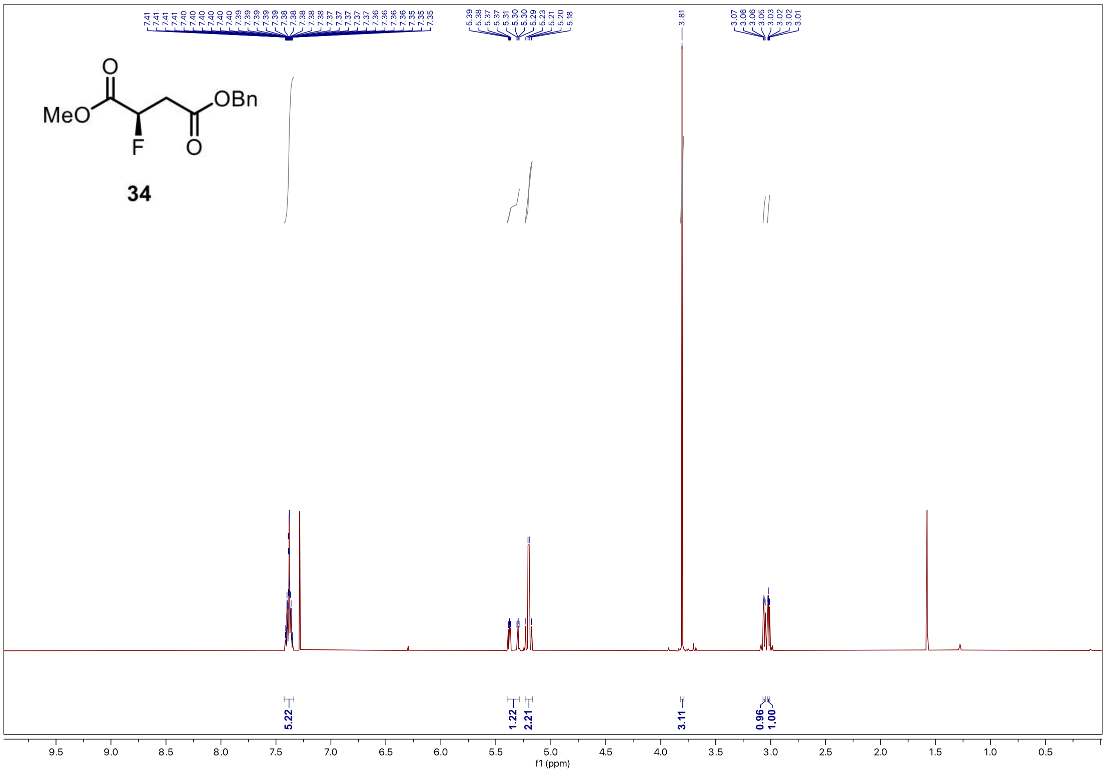


$^{19}F$ NMR (376 MHz, $CDCl_3$)

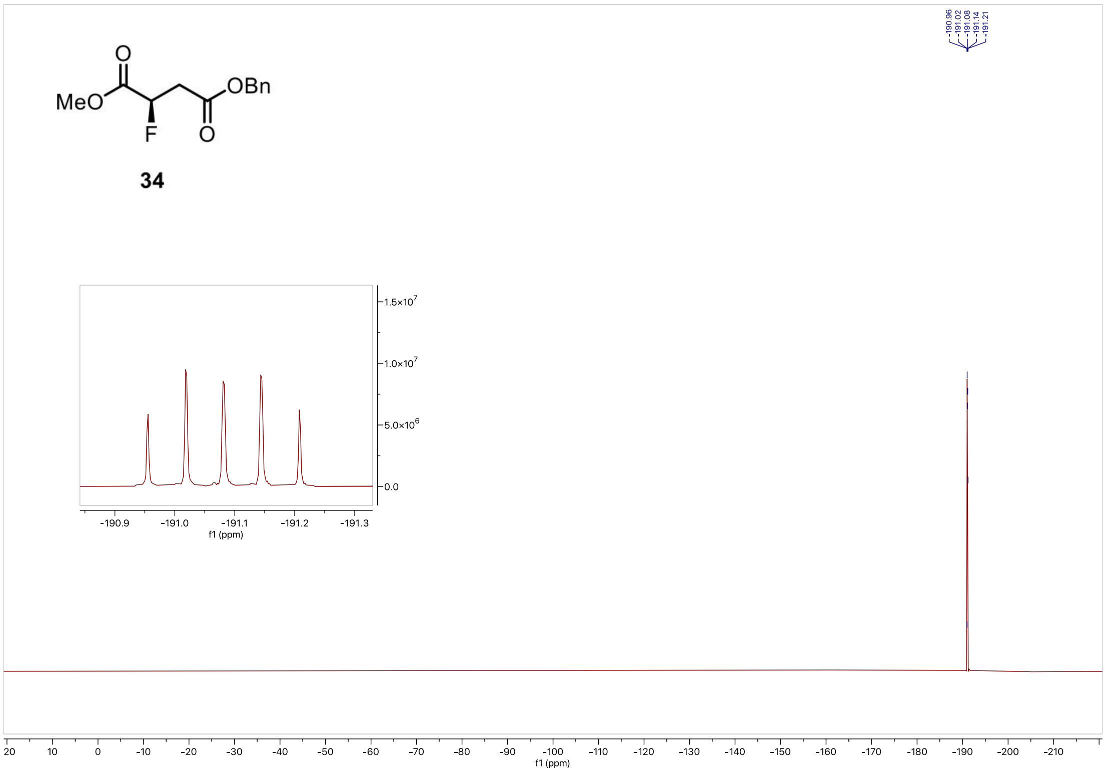

$^{13}C$ {1H} NMR (151 MHz, $CDCl_3$)

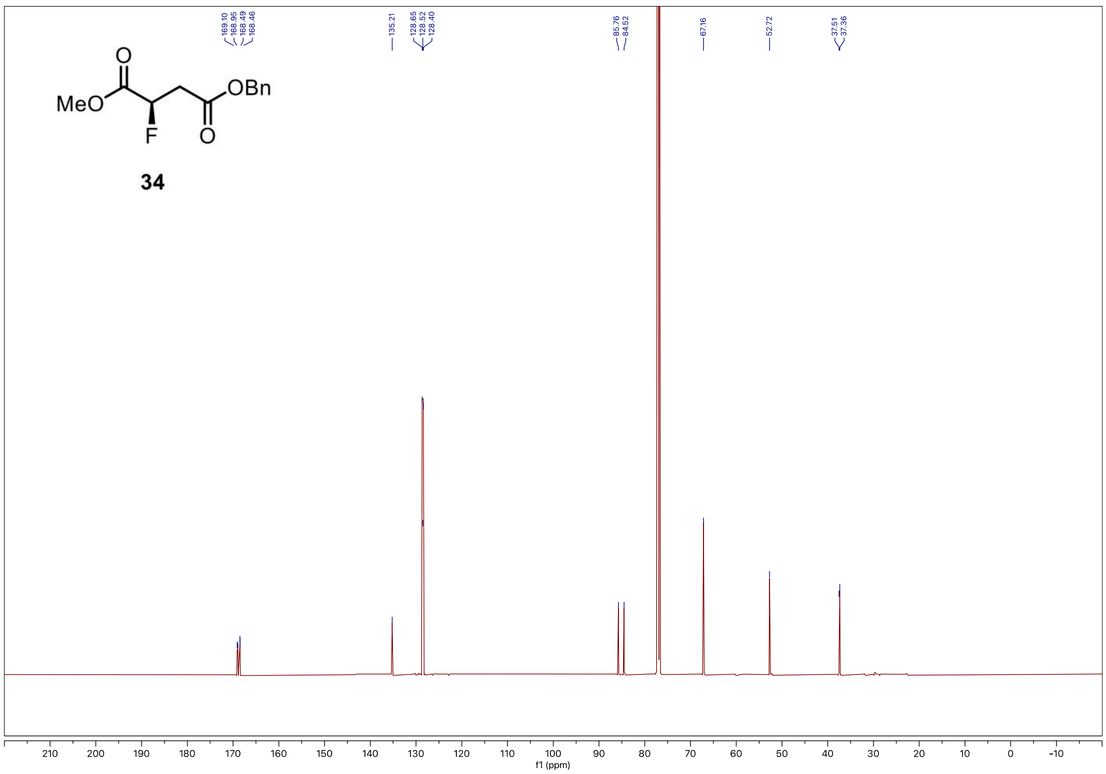

**Benzyl (*S*) -3-hydroxy-4-oxo-4-(*p*-tolylthio)butanoate (36):**

$^{1}$H NMR (600 MHz, $CDCl_3$)

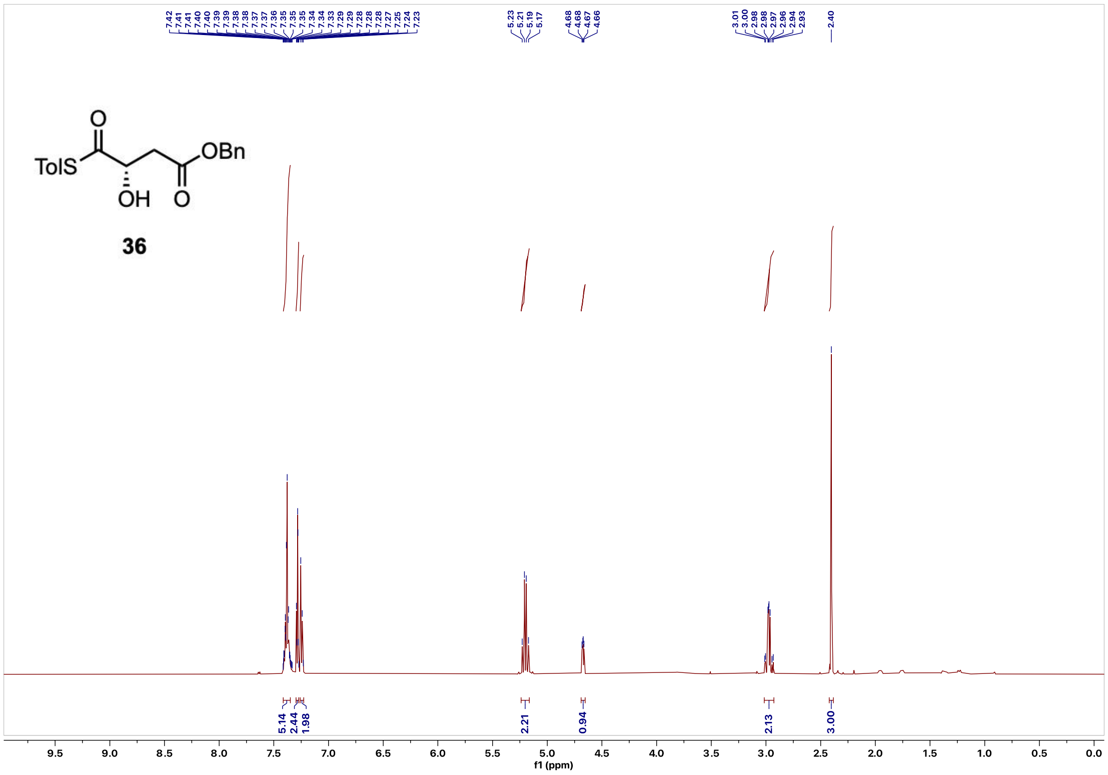

$^{13}$C{1H} NMR (151 MHz, $CDCl_3$)

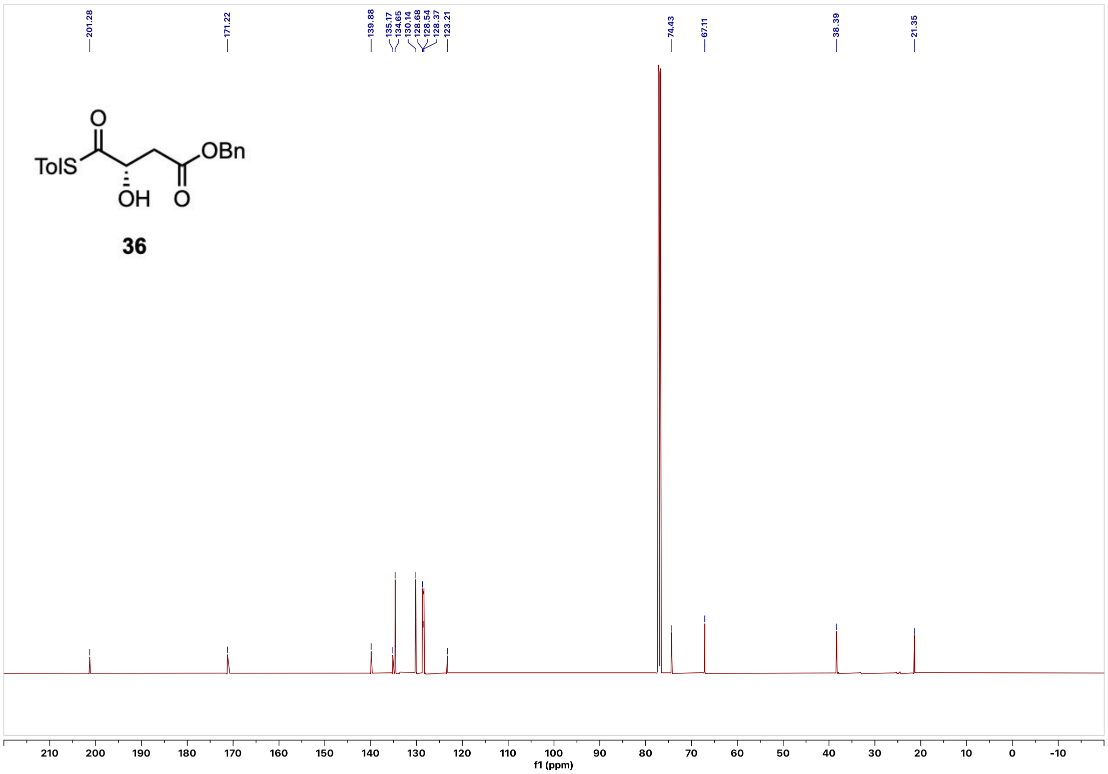

**Benzyl (*R*)-3-fluoro-4-oxo-4-(*p*-tolylthio)butanoate (35):**

$^1$H NMR (600 MHz, $CDCl_3$)

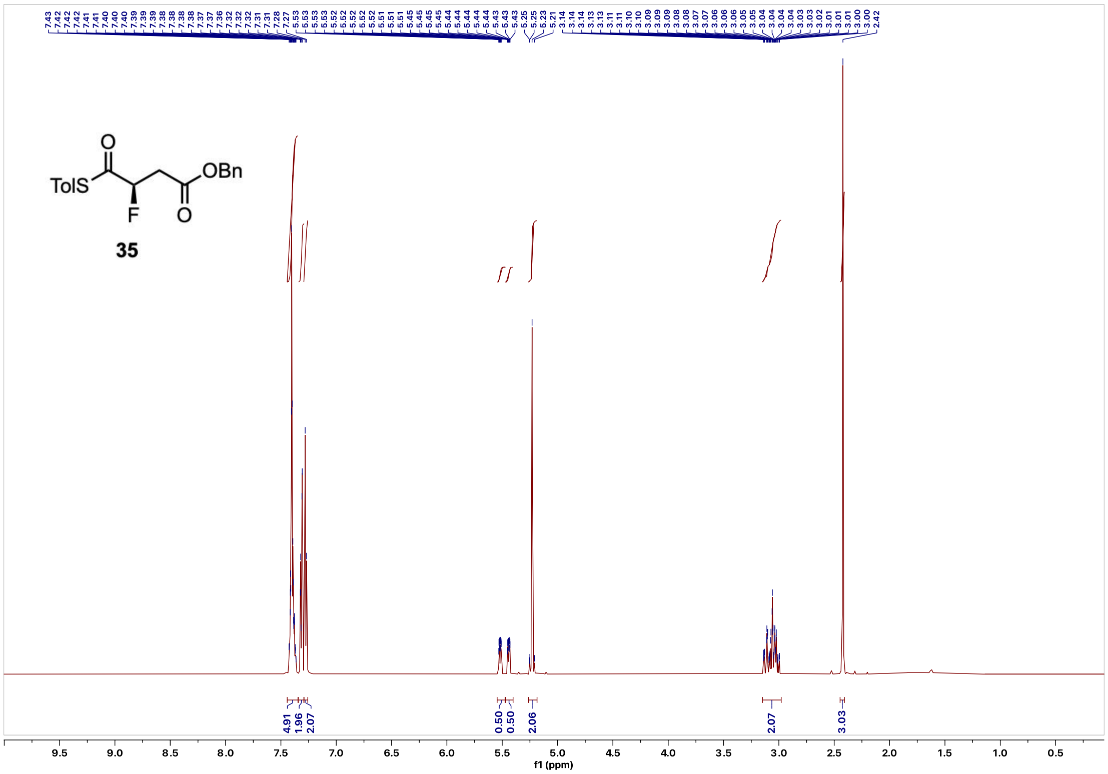


$^{19}$F NMR (565 MHz, CDCl3)

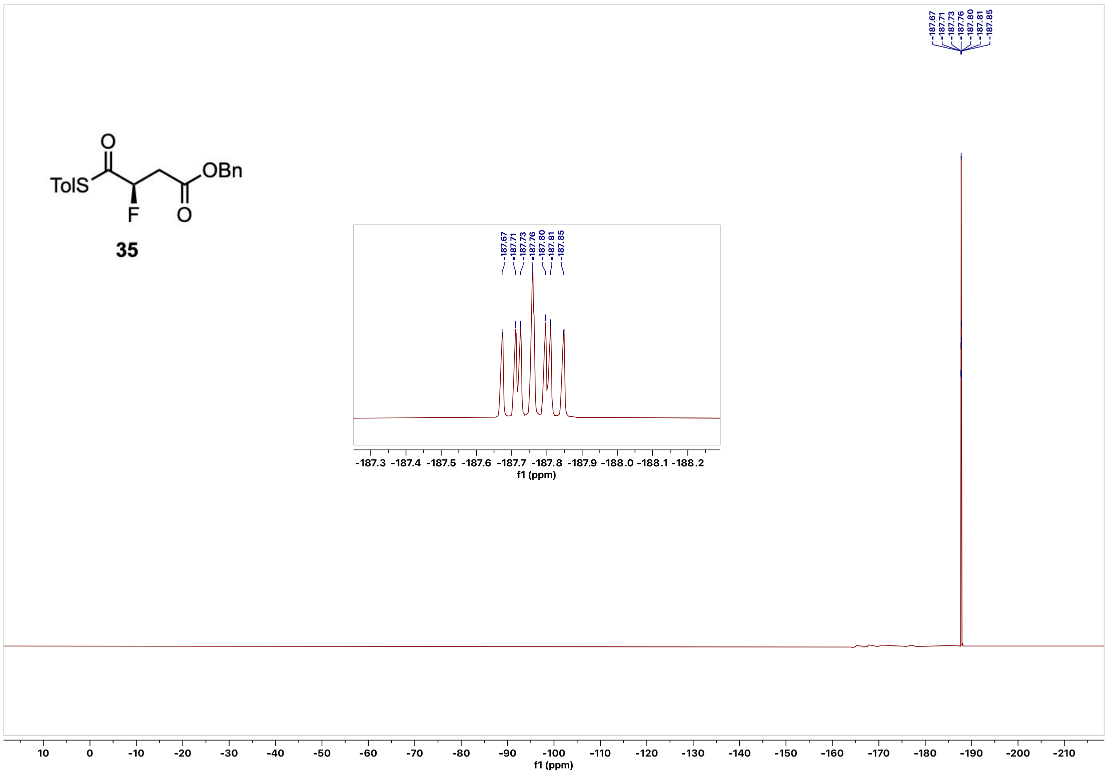

$^{13}C$ {1H} NMR (151 MHz, $CDCl_3$)

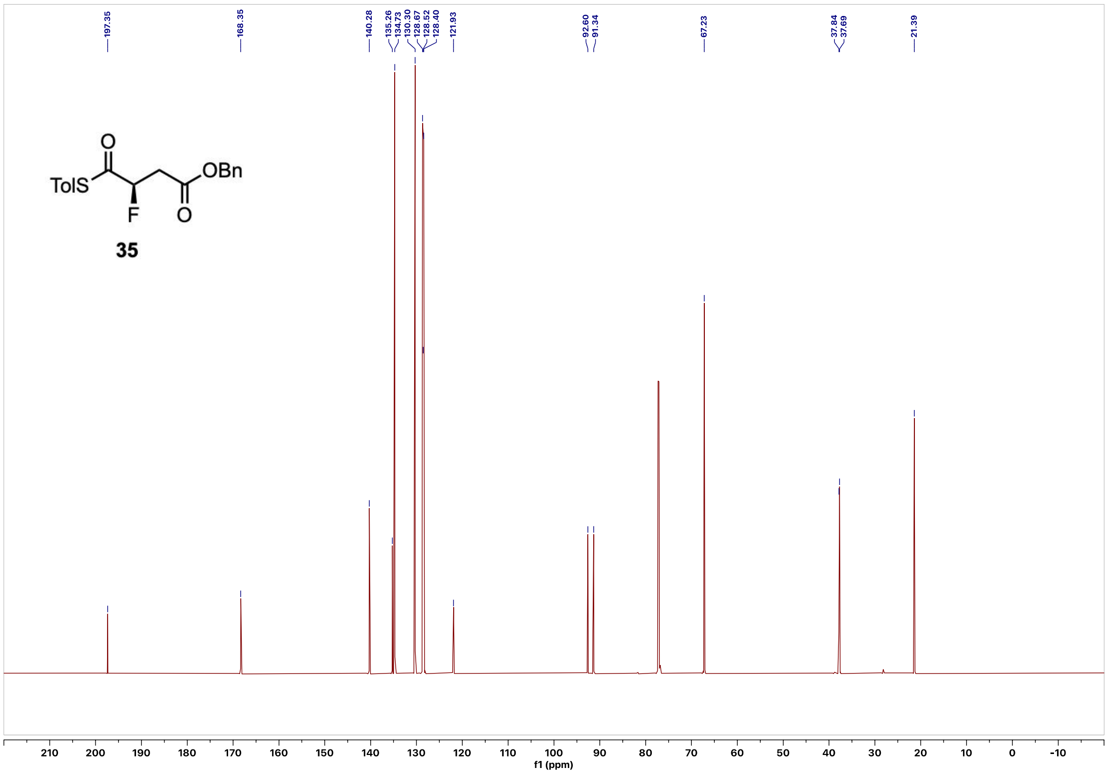

**Benzyl (*R*)-5-(((benzyloxy)carbonyl)amino)-3-fluoro-4-oxopentanoate (13a):**

$^{1}$H NMR (400 MHz, $CDCl_3$)

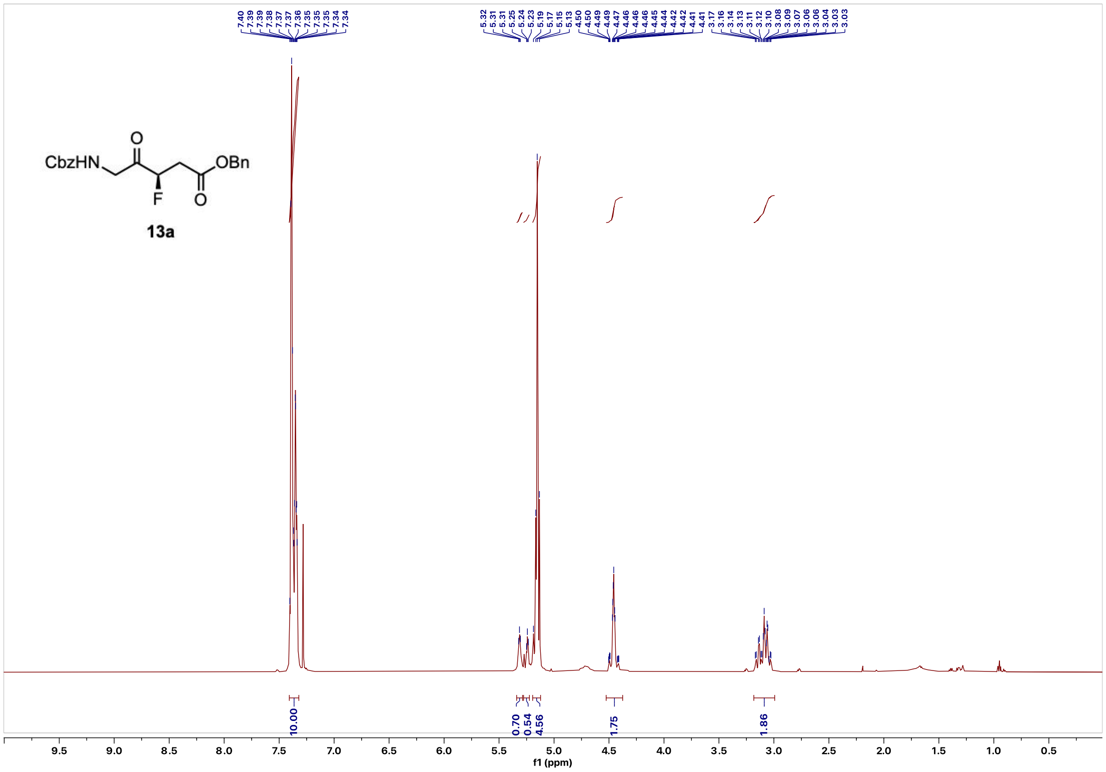

$^{19}$F NMR (376 MHz, $CDCl_3$)

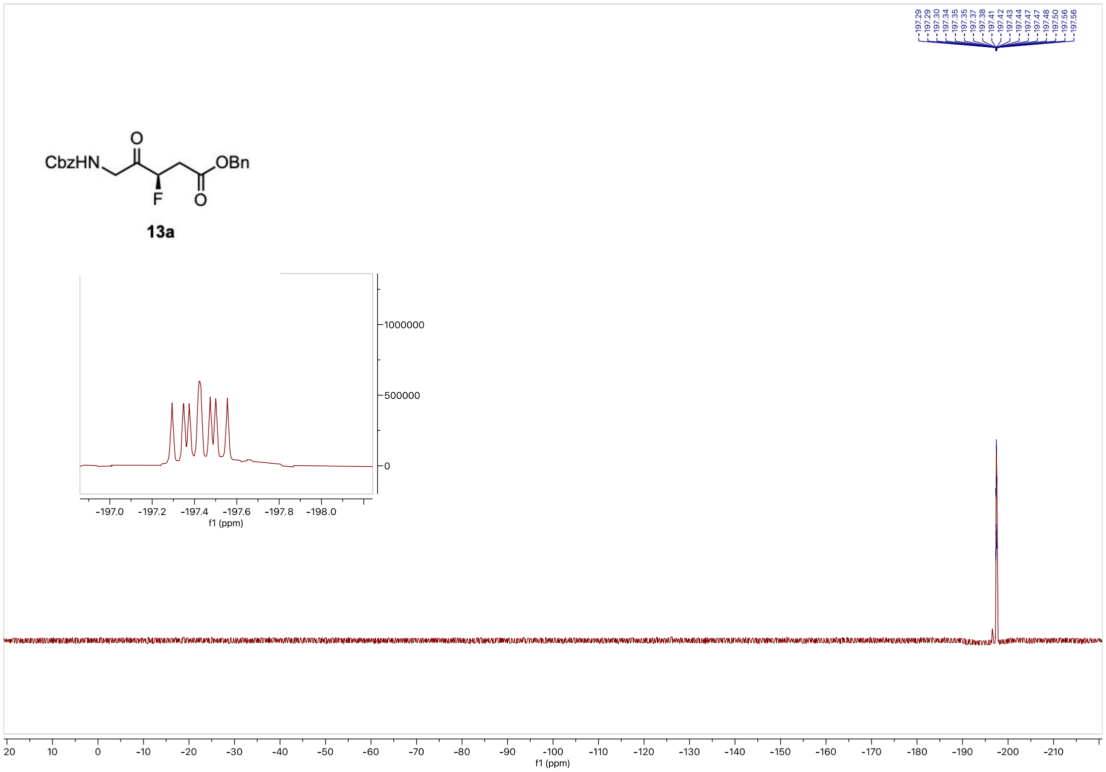

$^{13}C$ {1H} NMR (151 MHz, $CDCl_3$)

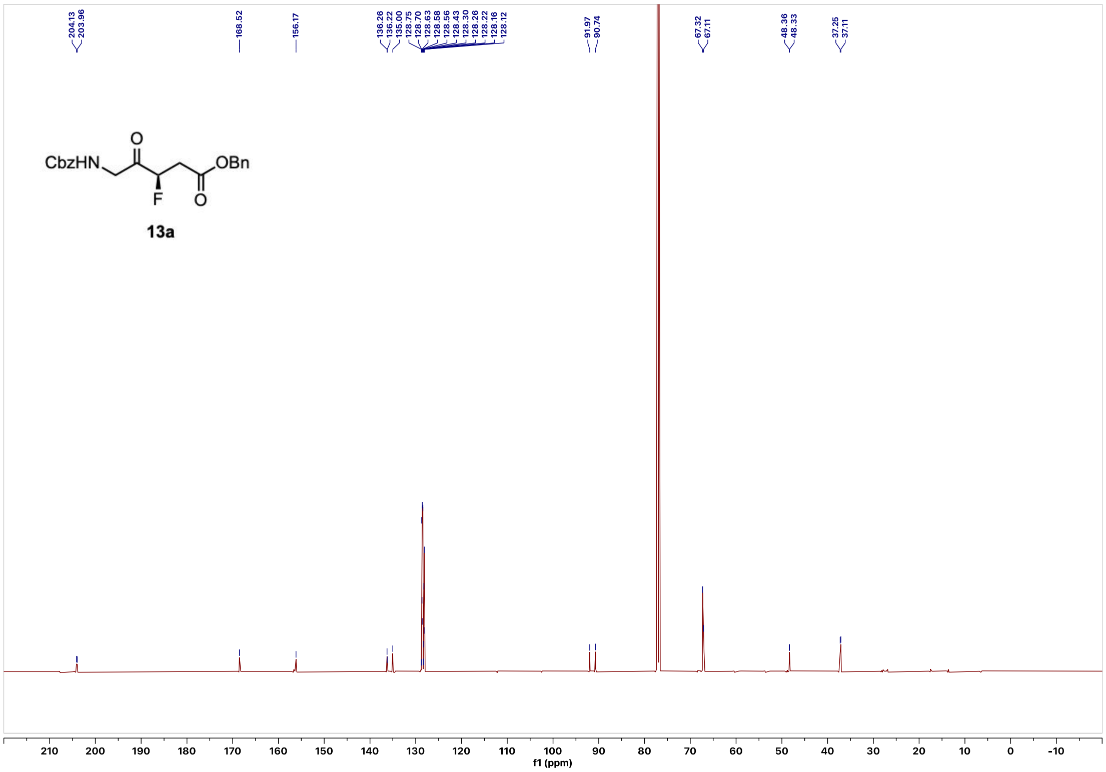

**(*R*)-5-amino-3-fluoro-4-oxopentanoic acid hydrochloride (14a):**

$^{1}H$ NMR (600 MHz, DMSO)

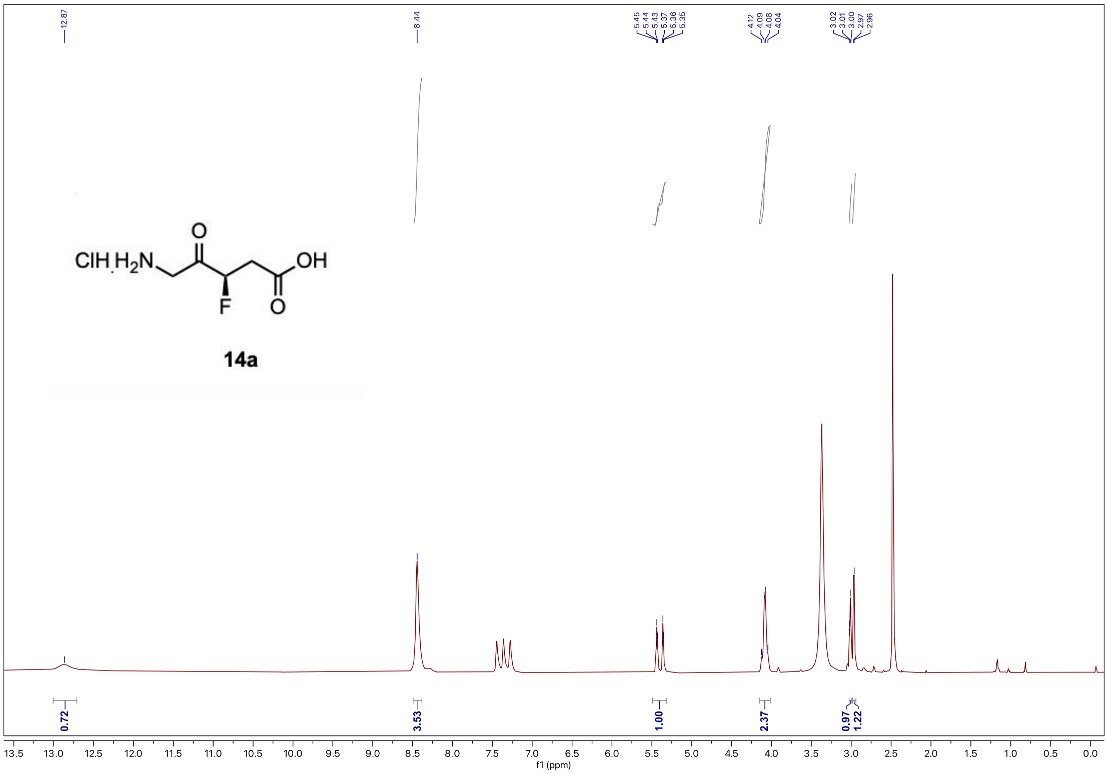


$^{19}F$ NMR (376 MHz, DMSO)

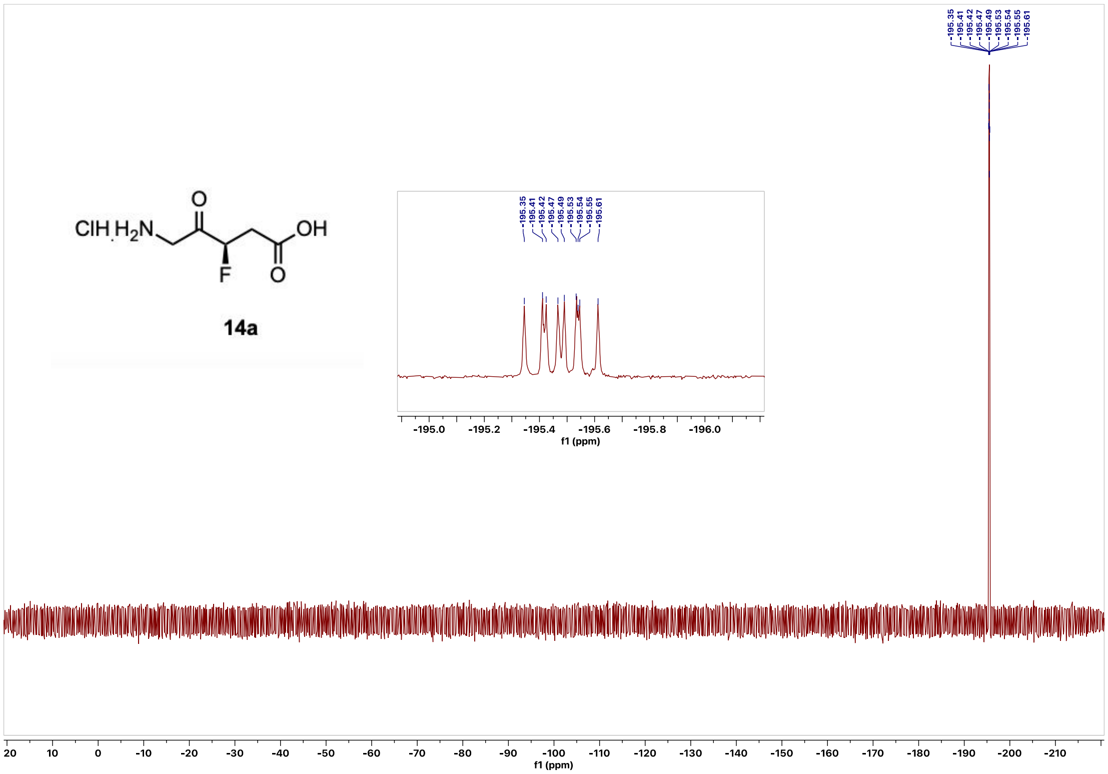

$^{13}C$ {1H} NMR (101 MHz, DMSO)

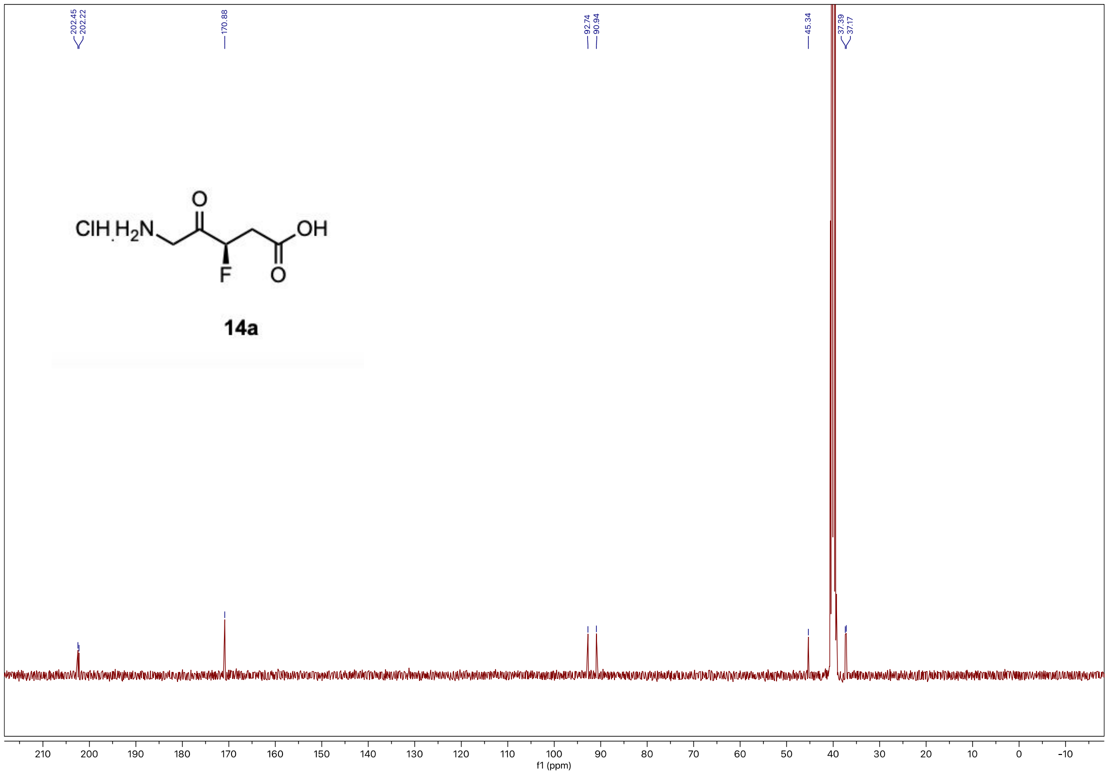

# II. X-ray crystallographic data for Compound *R* and *S*-3F-5-ALA

| Compound | R-3F-5-ALA |
|---|---|
| Formula | $C_5H_9ClFNO_3$ |
| $D_{calc.}$/ g cm$^{-3}$ | 1.519 |
| $\mu$/mm$^{-1}$ | 4.080 |
| Formula Weight | 185.583 |
| Color | colorless |
| Shape | needle-shaped |
| Size/mm$^3$ | 0.46×0.10×0.06 |
| *T*/K | 100.00(10) |
| Crystal System | monoclinic |
| Flack Parameter | -0.001(15) |
| Hooft Parameter | 0.007(7) |
| Space Group | $P2_1$ |
| *a*/Å | 8.2939(4) |
| *b*/Å | 5.5671(3) |
| *c*/Å | 8.9079(4) |
| $\alpha$/° | 90 |
| $\beta$/° | 99.376(4) |
| $\gamma$/° | 90 |
| V/Å$^3$ | 405.81(4) |
| *Z* | 2 |
| *Z'* | 1 |
| Wavelength/Å | 1.54184 |
| Radiation type | Cu $K_\alpha$ |
| $\Theta_{min}$/° | 5.03 |
| $\Theta_{max}$/° | 76.98 |
| Measured Refl's. | 4610 |
| Indep't Refl's | 1562 |
| Refl's I≥2 $\sigma$(I) | 1541 |
| $R_{int}$ | 0.0312 |
| Parameters | 181 |
| Restraints | 100 |
| Largest Peak | 0.1870 |
| Deepest Hole | -0.1869 |
| GooF | 1.1363 |
| $wR_2$ (all data) | 0.0742 |
| $wR_2$ | 0.0740 |
| $R_1$ (all data) | 0.0274 |
| $R_1$ | 0.0271 |

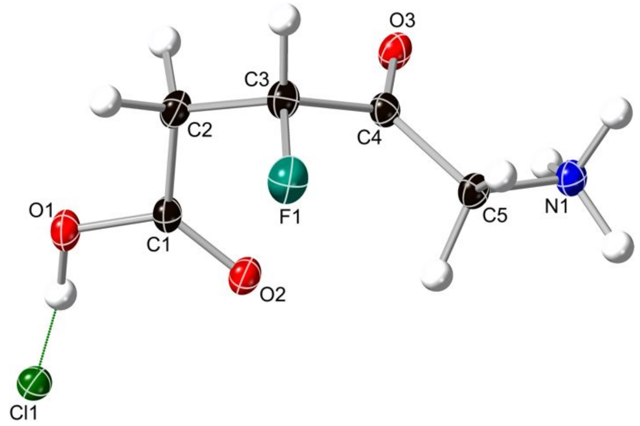


**Experimental.** Single colorless needle-shaped crystals of **R-3F-5-ALA** were chosen from the sample as supplied. A suitable crystal with dimensions 0.46 × 0.10 × 0.06 mm$^3$ was selected and mounted on a loop with paratone on a Rigaku Synergy-S diffractometer. The crystal was kept at a steady $T$ = 100.0(1) K during data collection. The structure was solved with the **ShelXT** 2018/2 (Sheldrick, 2018) solution program using dual methods and by using **Olex2** 1.3-alpha (Dolomanov et al., 2009) as the graphical interface. The model was refined with **olex2.refine** 1.3-alpha (Bourhis et al., 2015) using full matrix least squares minimisation on $\boldsymbol{F^2}$.

**Crystal Data.** $C_5H_9ClFNO_3$, $M_r$ = 185.583, monoclinic, $P2_1$ (No. 4), a = 8.2939(4) Å, b = 5.5671(3) Å, c = 8.9079(4) Å, $\beta$ = 99.376(4)°, $\alpha = \gamma$ = 90°, $V$ = 405.81(4) Å$^3$, $T$ = 100.00(10) K, $Z$ = 2, $Z'$ = 1, $\mu$(Cu K$_\alpha$) = 4.080, 4610 reflections measured, 1562 unique ($R_{int}$ = 0.0312) which were used in all calculations. The final $wR_2$ was 0.0742 (all data) and $R_1$ was 0.0271 (I≥2 $\sigma$(I)).

## Structure Quality Indicators

| | | | | | |
|---|---|---|---|---|---|
| **Reflections:** | d min (Cu\a) 2θ=154.0° 0.79 | I/σ(I) 36.4 | Rint 3.12% | CAP 130.0° 93% to 154.0° 99.7 | |
| **Refinement:** | Shift 0.001 | Max Peak 0.2 | Min Peak -0.2 | GooF 1.136 | Flack -.001(15) |

**Table 1**: Fractional Atomic Coordinates (×10$^4$) and Equivalent Isotropic Displacement Parameters (Å$^2$×10$^3$) for **R-3F-ALA**. $U_{eq}$ is defined as 1/3 of the trace of the orthogonalised $U_{ij}$.

| Atom | x | y | z | $U_{eq}$ |
|---|---|---|---|---|
| Cl1 | -1059.9(4) | 8841.6(8) | 8621.9(5) | 23.08(14) |
| F1 | 4205.1(13) | 2853(2) | 5254.9(11) | 27.6(3) |
| O1 | 69.4(14) | 4836(3) | 6897.0(15) | 22.7(3) |
| O3 | 4733.5(15) | 1158(2) | 9082.5(15) | 21.0(3) |
| O2 | 2710.2(16) | 5824(2) | 7585.3(16) | 23.4(3) |
| N1 | 7450.5(15) | 3774(4) | 9274.9(16) | 18.9(3) |
| C1 | 1661.3(19) | 4399(3) | 7061.4(18) | 17.8(4) |
| C5 | 6359.9(18) | 3701(4) | 7798.0(18) | 19.2(3) |
| C2 | 2004(2) | 1892(3) | 6549(2) | 19.5(4) |
| C4 | 4932(2) | 2064(3) | 7894.0(19) | 17.1(3) |
| C3 | 3778(2) | 1486(3) | 6441(2) | 19.0(4) |

**Table 2**: Anisotropic Displacement Parameters (×10$^4$) for **R-3F-ALA**. The anisotropic displacement factor exponent takes the form: $-2\pi^2[h^2a^{*2} \times U_{11} + ... + 2hka^* \times b^* \times U_{12}]$

| Atom | $U_{11}$ | $U_{22}$ | $U_{33}$ | $U_{23}$ | $U_{13}$ | $U_{12}$ |
|---|---|---|---|---|---|---|
| Cl1 | 17.3(2) | 16.7(2) | 35.1(2) | 0.76(16) | 3.85(14) | -2.42(17) |
| F1 | 25.2(6) | 38.2(6) | 20.1(5) | -3.8(5) | 5.5(4) | 2.0(5) |
| O1 | 13.2(6) | 25.3(6) | 28.9(6) | 0.5(5) | 1.0(4) | -3.4(5) |
| O3 | 16.8(6) | 24.9(6) | 21.6(6) | -5.1(5) | 3.5(5) | 4.6(5) |
| O2 | 15.7(6) | 20.3(7) | 33.4(7) | -1.7(5) | 1.7(5) | -4.5(5) |
| N1 | 13.4(6) | 20.2(7) | 23.5(7) | 0.2(8) | 4.2(5) | -0.9(7) |
| C1 | 12.6(7) | 20.7(10) | 19.8(8) | -0.1(6) | 1.7(5) | 0.1(6) |
| C5 | 13.8(7) | 21.3(9) | 22.7(8) | -1.6(8) | 4.2(5) | 2.8(8) |
| C2 | 15.1(8) | 20.9(9) | 22.2(9) | -2.9(7) | 2.1(7) | -4.7(7) |
| C4 | 13.4(8) | 19.3(7) | 18.9(8) | 0.4(7) | 3.7(6) | 1.1(6) |
| C3 | 15.9(8) | 23.1(10) | 18.1(8) | -0.6(7) | 3.3(6) | -2.0(7) |
| H1A | 25(15) | 26(8) | 50(20) | 8(5) | -7(13) | -10(9) |
| H1B | 18(5) | 20(5) | 24(4) | -0.1(17) | 5.7(18) | -0.9(15) |
| H1C | 60(19) | 44(9) | 35(18) | -33(7) | -13(14) | 12(10) |
| H5A | 35(14) | 50(20) | 37(13) | 6(11) | 18(6) | -2(9) |
| H2A | 30(15) | 38(16) | 38(15) | 2(11) | 20(10) | -12(10) |
| H3 | 63(19) | 30(14) | 29(15) | 2(10) | 6(13) | -12(9) |
| H5B | 31(16) | 26(7) | 90(20) | 2(5) | -8(15) | 14(7) |
| H2B | 53(18) | 26(17) | 21(13) | -4(12) | -1(9) | -12(9) |
| H1 | 20(17) | 25(17) | 40(20) | 7(11) | -2(14) | -11(11) |

**Table 3**: Bond Lengths in Å for **R-3F-ALA**.

| Atom | Atom | Length/Å |
|---|---|---|
| F1 | C3 | 1.394(2) |
| O1 | C1 | 1.327(2) |
| O3 | C4 | 1.208(2) |
| O2 | C1 | 1.213(2) |
| N1 | C5 | 1.471(2) |
| C1 | C2 | 1.509(2) |
| C5 | C4 | 1.508(2) |
| C2 | C3 | 1.508(2) |
| C4 | C3 | 1.513(2) |

**Table 4**: Bond Angles in ˚ for **R-3F-ALA**.

| Atom | Atom | Atom | Angle/˚ |
|---|---|---|---|
| O2 | C1 | O1 | 124.10(16) |
| C2 | C1 | O1 | 111.66(14) |
| C2 | C1 | O2 | 124.22(15) |
| C4 | C5 | N1 | 109.90(14) |
| C3 | C2 | C1 | 113.06(15) |
| C5 | C4 | O3 | 121.49(15) |
| C3 | C4 | O3 | 120.24(16) |
| C3 | C4 | C5 | 118.22(14) |
| C2 | C3 | F1 | 109.75(14) |
| C4 | C3 | F1 | 109.21(14) |
| C4 | C3 | C2 | 113.64(15) |

**Table 5**: Torsion Angles in ˚ for **R-3F-ALA**.

| Atom | Atom | Atom | Atom | Angle/˚ |
|---|---|---|---|---|
| F1 | C3 | C2 | C1 | -67.99(15) |
| F1 | C3 | C4 | O3 | 176.54(14) |
| F1 | C3 | C4 | C5 | -6.07(17) |
| O1 | C1 | C2 | C3 | 169.21(14) |
| O3 | C4 | C5 | N1 | 5.12(19) |
| O3 | C4 | C3 | C2 | 53.65(19) |

| Atom | Atom | Atom | Atom | Angle/˚ |
|---|---|---|---|---|
| O2 | C1 | C2 | C3 | -12.07(19) |
| N1 | C5 | C4 | C3 | -172.24(15) |
| C1 | C2 | C3 | C4 | 54.60(17) |
| C5 | C4 | C3 | C2 | -128.96(17) |

**Table 6**: Hydrogen Fractional Atomic Coordinates (×10$^4$) and Equivalent Isotropic Displacement Parameters (Å$^2$×10$^3$) for **R-3F-ALA**. $U_{eq}$ is defined as 1/3 of the trace of the orthogonalised $U_{ij}$.

| Atom | x | y | z | $U_{eq}$ |
|---|---|---|---|---|
| H1A | 8170(40) | 2300(70) | 9370(40) | 34(8) |
| H1B | 6770(30) | 3790(60) | 10160(30) | 20(4) |
| H1C | 8140(50) | 5250(70) | 9340(40) | 49(9) |
| H5A | 7080(30) | 3040(60) | 6940(30) | 39(8) |
| H2A | 1590(30) | 690(60) | 7350(30) | 34(7) |
| H3 | 3920(40) | -390(60) | 6140(30) | 41(7) |
| H5B | 5840(40) | 5490(60) | 7450(50) | 51(9) |
| H2B | 1230(30) | 1440(60) | 5480(30) | 34(7) |
| H1 | -210(40) | 6420(80) | 7410(40) | 30(9) |

**Table 7**: Hydrogen Bond information for **R-3F-ALA**.

| D | H | A | d(D-H)/Å | d(H-A)/Å | d(D-A)/Å | D-H-A/deg |
|---|---|---|---|---|---|---|
| N1 | H1B | O3[1] | 1.04(2) | 2.00(3) | 2.837(2) | 135(3) |

[1]1-x,1/2+y,2-z

**Table 8**: Selected Bond Lengths in Å for **R-3F-ALA**.

| Atom | Atom | Length/Å |
|---|---|---|
| O1 | H1 | 1.03(4) |
| N1 | H1A | 1.01(4) |
| N1 | H1B | 1.04(2) |
| N1 | H1C | 1.00(4) |
| C5 | H5A | 1.11(3) |
| C5 | H5B | 1.11(3) |
| C2 | H2A | 1.08(3) |
| C2 | H2B | 1.09(3) |
| C3 | H3 | 1.09(3) |

**Table 9**: Selected Bond Angles in ˚ for **R-3F-ALA**.

| Atom | Atom | Atom | Angle/˚ |
|---|---|---|---|
| H1 | O1 | C1 | 113.4(18) |
| H1A | N1 | C5 | 109(2) |
| H1B | N1 | C5 | 110.3(13) |
| H1B | N1 | H1A | 109(3) |
| H1C | N1 | C5 | 110(2) |
| H1C | N1 | H1A | 110(3) |
| H1C | N1 | H1B | 109(3) |
| H5A | C5 | N1 | 107.8(15) |
| H5A | C5 | C4 | 110.8(16) |
| H5B | C5 | N1 | 112.3(19) |
| H5B | C5 | C4 | 106.5(16) |
| H5B | C5 | H5A | 110(3) |
| H2A | C2 | C1 | 106.2(15) |
| H2A | C2 | C3 | 111.6(15) |
| H2B | C2 | C1 | 111.3(17) |
| H2B | C2 | C3 | 110.7(15) |
| H2B | C2 | H2A | 104(2) |
| H3 | C3 | F1 | 106.7(15) |
| H3 | C3 | C2 | 107.8(16) |
| H3 | C3 | C4 | 109.5(15) |

| **Compound** | **S-3F-5-ALA** |
|---|---|
| Formula | $C_5H_9ClFNO_3$ |
| $D_{calc.}$/ g cm$^{-3}$ | 1.518 |
| $\mu$/mm$^{-1}$ | 4.077 |
| Formula Weight | 185.583 |
| Color | colorless |
| Shape | needle-shaped |
| Size/mm$^3$ | 0.36×0.07×0.05 |
| $T$/K | 100.00(10) |
| Crystal System | monoclinic |
| Flack Parameter | 0.013(16) |
| Hooft Parameter | 0.004(8) |
| Space Group | $P2_1$ |
| $a$/Å | 8.2907(3) |
| $b$/Å | 5.57432(19) |
| $c$/Å | 8.9064(4) |
| $\alpha$/° | 90 |
| $\beta$/° | 99.350(4) |
| $\gamma$/° | 90 |
| V/Å$^3$ | 406.14(3) |
| $Z$ | 2 |
| $Z'$ | 1 |
| Wavelength/Å | 1.54184 |
| Radiation type | Cu $K_\alpha$ |
| $\Theta_{min}$/° | 5.03 |
| $\Theta_{max}$/° | 72.95 |
| Measured Refl's. | 8457 |
| Indep't Refl's | 1546 |
| Refl's I≥2 $\sigma$(I) | 1530 |
| $R_{\mathrm{int}}$ | 0.0516 |
| Parameters | 181 |
| Restraints | 103 |
| Largest Peak | 0.2159 |
| Deepest Hole | -0.1318 |
| GooF | 1.0985 |
| $wR_2$ (all data) | 0.0621 |
| $wR_2$ | 0.0619 |
| $R_1$ (all data) | 0.0248 |
| $R_1$ | 0.0244 |

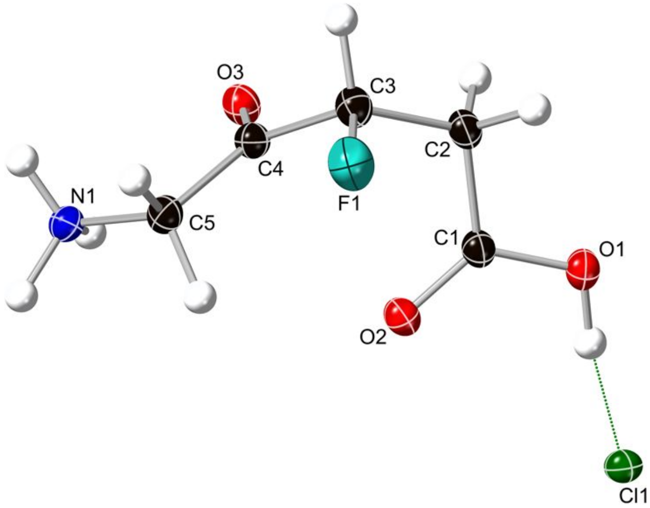


**Experimental.** Single colorless needle-shaped crystals of **S-3F-ALA** were chosen from the sample as supplied. A suitable crystal with dimensions 0.36 × 0.07 × 0.05 mm$^3$ was selected and mounted on a loop with paratone on a XtaLAB Synergy-S diffractometer. The crystal was kept at a steady $T$ = 100.0(1) K during data collection. The structure was solved with the **ShelXT** 2018/2 (Sheldrick, 2018) solution program using dual methods and by using **Olex2** 1.3-alpha (Dolomanov et al., 2009) as the graphical interface. The model was refined with **olex2.refine** 1.3-alpha (Bourhis et al., 2015) using full matrix least squares minimisation on $\boldsymbol{F^2}$.

**Crystal Data.** $C_5H_9ClFNO_3$, $M_r$ = 185.583, monoclinic, $P2_1$ (No. 4), a = 8.2907(3) Å, b = 5.57432(19) Å, c = 8.9064(4) Å, $\beta$ = 99.350(4)°, $\alpha = \gamma$ = 90°, $V$ = 406.14(3) Å$^3$, $T$ = 100.00(10) K, $Z$ = 2, $Z'$ = 1, $\mu$(Cu K$_\alpha$) = 4.077, 8457 reflections measured, 1546 unique ($R_{int}$ = 0.0516) which were used in all calculations. The final $wR_2$ was 0.0621 (all data) and $R_1$ was 0.0244 (I≥2 $\sigma$(I)).

## Structure Quality Indicators

| | | | | |
|---|---|---|---|---|
| **Reflections:** | d min (Cu\a) 2θ=145.9° 0.81 | I/σ(I) 33.6 | Rint 5.16% | CAP 130.0° 97% to 145.9° 100 |
| **Refinement:** | Shift 0.001 | Max Peak 0.2 | Min Peak -0.1 | GooF 1.099 | Flack .013(16) |

## Images of the Crystal on the Diffractometer

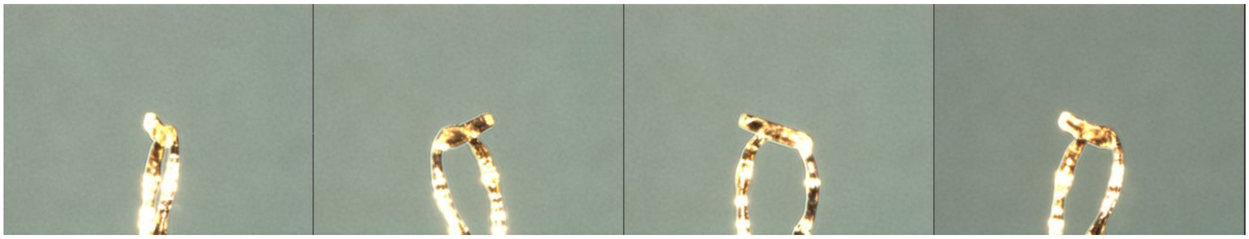

**Table 10**: Fractional Atomic Coordinates (×10$^4$) and Equivalent Isotropic Displacement Parameters (Å$^2$×10$^3$) for **S-3F-ALA**. $U_{eq}$ is defined as 1/3 of the trace of the orthogonalised $U_{ij}$.

| Atom | x | y | z | $U_{eq}$ |
|---|---|---|---|---|
| Cl1 | 1065.9(4) | -8836.8(8) | -8622.6(5) | 25.23(12) |
| F1 | -4203.7(13) | -2842(2) | -5257.5(12) | 32.4(3) |
| O1 | -67.7(14) | -4831(2) | -6899.7(15) | 25.3(3) |

| Atom | x | y | z | $U_{eq}$ |
|---|---|---|---|---|
| O3 | -4732.7(14) | -1168(2) | -9083.3(15) | 23.5(3) |
| O2 | -2710.8(15) | -5832(2) | -7581.0(16) | 26.7(3) |
| N1 | -7455.2(15) | -3772(3) | -9276.6(16) | 21.0(3) |
| C1 | -1656.8(18) | -4404(3) | -7059.6(19) | 20.3(4) |
| C5 | -6359.7(18) | -3707(4) | -7799.6(19) | 21.9(3) |
| C2 | -2004(2) | -1906(3) | -6547(2) | 22.2(4) |
| C4 | -4935.7(19) | -2071(3) | -7898.4(19) | 19.4(3) |
| C3 | -3782(2) | -1486(3) | -6440(2) | 23.0(4) |

**Table 11**: Anisotropic Displacement Parameters (×10[4]) for **S-3F-ALA**. The anisotropic displacement factor exponent takes the form: $-2\pi^2[h^2a^{*2} \times U_{11} + ... +2hka^* \times b^* \times U_{12}]$

| Atom | $U_{11}$ | $U_{22}$ | $U_{33}$ | $U_{23}$ | $U_{13}$ | $U_{12}$ |
|---|---|---|---|---|---|---|
| Cl1 | 20.77(18) | 17.27(19) | 37.6(2) | 0.61(17) | 4.72(14) | -2.65(19) |
| F1 | 30.6(6) | 42.3(6) | 25.4(6) | -4.6(5) | 7.2(4) | 2.0(5) |
| O1 | 17.6(5) | 26.8(6) | 30.5(7) | 1.1(5) | 0.8(5) | -3.4(5) |
| O3 | 21.0(6) | 25.0(6) | 24.6(7) | -4.7(5) | 4.5(5) | 4.3(5) |
| O2 | 19.6(6) | 23.1(7) | 36.9(8) | -2.6(5) | 2.6(5) | -4.9(5) |
| N1 | 16.9(6) | 20.1(6) | 26.8(7) | 1.8(8) | 5.5(5) | -1.5(8) |
| C1 | 16.8(7) | 22.1(10) | 22.0(8) | -1.1(6) | 2.6(6) | -1.0(7) |
| C5 | 17.8(7) | 22.1(8) | 26.6(9) | -0.9(8) | 5.5(6) | 4.9(9) |
| C2 | 17.4(8) | 24.3(9) | 24.3(10) | -2.6(7) | 1.9(7) | -3.3(8) |
| C4 | 17.5(8) | 19.9(7) | 21.7(9) | -0.8(7) | 6.4(7) | 0.0(7) |
| C3 | 19.4(8) | 27.0(10) | 23.3(9) | -0.4(7) | 5.5(7) | -4.8(7) |
| H1a | 64(18) | 47(10) | 42(18) | 37(6) | 6(14) | -3(10) |
| H1b | 25(10) | 63(18) | 34(7) | -26(14) | 13(4) | -25(11) |
| H1c | 34(15) | 29(7) | 60(20) | -10(5) | 0(13) | 3(9) |
| H5a | 22(10) | 40(16) | 38(11) | -9(8) | 12(5) | -10(7) |
| H2a | 32(15) | 70(20) | 60(18) | -17(12) | 6(11) | 23(12) |
| H3 | 83(19) | 18(12) | 54(18) | 17(9) | 21(15) | -15(9) |
| H5b | 44(15) | 26(5) | 56(17) | 7(4) | 5(13) | 11(5) |
| H2b | 42(15) | 26(15) | 36(13) | 3(11) | -8(9) | -4(9) |
| H1 | 24(5) | 26(3) | 27(5) | 1.4(12) | 3.9(18) | -0.8(14) |

**Table 12**: Bond Lengths in Å for **S-3F-ALA**.

| Atom | Atom | Length/Å |
|---|---|---|
| F1 | C3 | 1.387(2) |
| O1 | C1 | 1.3236(19) |
| O3 | C4 | 1.205(2) |
| O2 | C1 | 1.217(2) |
| N1 | C5 | 1.473(2) |
| C1 | C2 | 1.507(3) |
| C5 | C4 | 1.506(2) |
| C2 | C3 | 1.511(2) |
| C4 | C3 | 1.518(2) |

**Table 13**: Bond Angles in ˚ for **S-3F-ALA**.

| Atom | Atom | Atom | Angle/˚ |
|---|---|---|---|
| O2 | C1 | O1 | 124.31(16) |
| C2 | C1 | O1 | 111.67(14) |
| C2 | C1 | O2 | 124.01(14) |
| C4 | C5 | N1 | 109.79(15) |
| C3 | C2 | C1 | 113.48(15) |
| C5 | C4 | O3 | 121.76(16) |

| Atom | Atom | Atom | Angle/˚ |
|---|---|---|---|
| C3 | C4 | O3 | 120.08(15) |
| C3 | C4 | C5 | 118.11(15) |
| C2 | C3 | F1 | 109.38(15) |

| Atom | Atom | Atom | Angle/˚ |
|---|---|---|---|
| C4 | C3 | F1 | 109.36(14) |
| C4 | C3 | C2 | 113.49(15) |

**Table 14**: Torsion Angles in ˚ for **S-3F-ALA**.

| Atom | Atom | Atom | Atom | Angle/˚ |
|---|---|---|---|---|
| F1 | C3 | C2 | C1 | 68.21(16) |
| F1 | C3 | C4 | O3 | -176.45(14) |
| F1 | C3 | C4 | C5 | 6.15(18) |
| O1 | C1 | C2 | C3 | -169.62(15) |
| O3 | C4 | C5 | N1 | -5.52(18) |
| O3 | C4 | C3 | C2 | -54.03(19) |
| O2 | C1 | C2 | C3 | 11.6(2) |
| N1 | C5 | C4 | C3 | 171.83(15) |
| C1 | C2 | C3 | C4 | -54.20(17) |
| C5 | C4 | C3 | C2 | 128.58(17) |

**Table 15**: Hydrogen Fractional Atomic Coordinates (×10$^4$) and Equivalent Isotropic Displacement Parameters (Å$^2$×10$^3$) for **S-3F-ALA**. $U_{eq}$ is defined as 1/3 of the trace of the orthogonalised $U_{ij}$.

| Atom | x | y | z | $U_{eq}$ |
|---|---|---|---|---|
| H1a | -8130(40) | -2420(60) | -9320(40) | 51(9) |
| H1b | -6830(30) | -3850(60) | -10120(30) | 40(7) |
| H1c | -8160(40) | -5320(60) | -9350(40) | 41(8) |
| H5a | -7090(20) | -3070(40) | -6970(30) | 33(6) |
| H2a | -1600(30) | -710(60) | -7320(40) | 54(9) |
| H3 | -4020(30) | 370(40) | -6270(30) | 51(7) |
| H5b | -5920(30) | -5500(50) | -7500(30) | 42(7) |
| H2b | -1230(30) | -1570(50) | -5490(30) | 36(6) |
| H1 | 160(30) | -6350(60) | -7320(30) | 26(4) |

**Table 16**: Selected Bond Lengths in Å for **S-3F-ALA**.

| Atom | Atom | Length/Å |
|---|---|---|
| O1 | H1 | 0.96(3) |
| N1 | H1a | 0.94(3) |
| N1 | H1b | 0.98(2) |
| N1 | H1c | 1.04(3) |
| C5 | H5a | 1.09(2) |

| Atom | Atom | Length/Å |
|---|---|---|
| C5 | H5b | 1.08(3) |
| C2 | H2a | 1.05(3) |
| C2 | H2b | 1.07(2) |
| C3 | H3 | 1.07(2) |

**Table 17**: Selected Bond Angles in ˚ for **S-3F-ALA**.

| Atom | Atom | Atom | Angle/˚ |
|---|---|---|---|
| H1 | O1 | C1 | 112.0(16) |
| H1a | N1 | C5 | 107(2) |
| H1b | N1 | C5 | 111.2(13) |
| H1b | N1 | H1a | 113(3) |

| Atom | Atom | Atom | Angle/˚ |
|---|---|---|---|
| H1c | N1 | C5 | 110(2) |
| H1c | N1 | H1a | 110(2) |
| H1c | N1 | H1b | 106(3) |
| H5a | C5 | N1 | 106.7(11) |

| Atom | Atom | Atom | Angle/˚ |
|---|---|---|---|
| H5a | C5 | C4 | 111.7(12) |
| H5b | C5 | N1 | 109.2(15) |
| H5b | C5 | C4 | 109.9(14) |
| H5b | C5 | H5a | 109(2) |
| H2a | C2 | C1 | 106.7(19) |
| H2a | C2 | C3 | 111.1(15) |
| H2b | C2 | C1 | 108.3(14) |
| H2b | C2 | C3 | 111.7(13) |
| H2b | C2 | H2a | 105(2) |
| H3 | C3 | F1 | 110.0(15) |
| H3 | C3 | C2 | 111.3(16) |
| H3 | C3 | C4 | 103.2(16) |

## III. Copies of HPLC chromatograms of compounds 36, 35 and 13a

HPLC chromatogram of compound **36** (Chiralpak IA, 1 mL.min$^{-1}$, Hex/*i*-PrOH = 95:5)

On top: mixture of (*R*) and (*S*) enantiomers; below: compound **36**

```
Acq. Operator    : SYSTEM                         Seq. Line :   2
Sample Operator  : SYSTEM
Acq. Instrument  : Normal Phase                    Location :   P2-C-03
Injection Date   : 6/17/2024 2:56:50 PM                 Inj :   1
                                                Inj Volume : 5.000 µl
Method           : C:\Users\Public\Documents\ChemStation\1\Data\CHIRAL_SCREENS\2024-06-17 14-45-
                   25 10%IPA_SCREEN_30min\10_IPA_IA_FIXED_1.0mL-min_30min.M (Sequence Method)
Last changed     : 10/1/2022 2:18:02 PM by SYSTEM
Method Info      : Date Created: 09/27/2022
                   Created By: Patrick Gross
                   %IPA: 10
                   Column: IA
                   Flowrate: 1.0 mL/min
                   Runtime: 30.0 min
                   Wavelengths: 210nm, 230nm, 254nm, 280nm

Additional Info : Peak(s) manually integrated
```

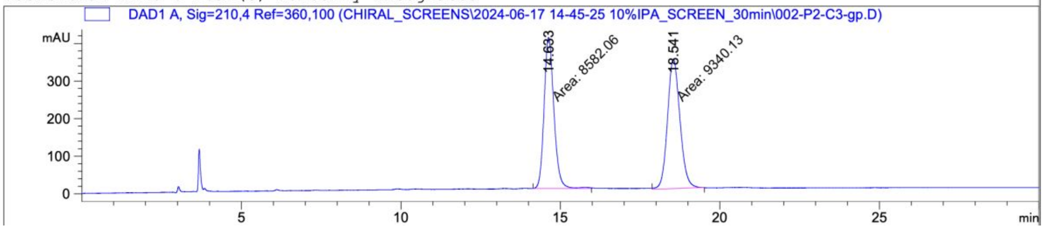


| Peak # | RetTime [min] | Type | Width [min] | Area [mAU*s] | Height [mAU] | Area % |
|---|---|---|---|---|---|---|
| 1 | 14.633 | MM | 0.3561 | 8582.05762 | 401.62094 | 47.8851 |
| 2 | 18.541 | MM | 0.4520 | 9340.13281 | 344.40918 | 52.1149 |

```
Acq. Operator    : SYSTEM                         Seq. Line :   2
Sample Operator  : SYSTEM
Acq. Instrument  : Normal Phase                    Location :   P2-C-04
Injection Date   : 6/17/2024 5:51:21 PM                 Inj :   1
                                                Inj Volume : 5.000 µl
Method           : C:\Users\Public\Documents\ChemStation\1\Data\IA_NP_Data\2024-06-17 17-39-58 10
                   %IPA_IA_30min\10_IPA_IA_FIXED_1.0mL-min_30min.M (Sequence Method)
Last changed     : 10/1/2022 2:18:02 PM by SYSTEM
Method Info      : Date Created: 09/27/2022
                   Created By: Patrick Gross
                   %IPA: 10
                   Column: IA
                   Flowrate: 1.0 mL/min
                   Runtime: 30.0 min
                   Wavelengths: 210nm, 230nm, 254nm, 280nm
```

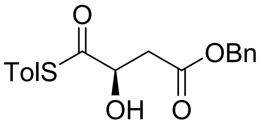


**36 (*R* isomer)**

```
Additional Info : Peak(s) manually integrated
```

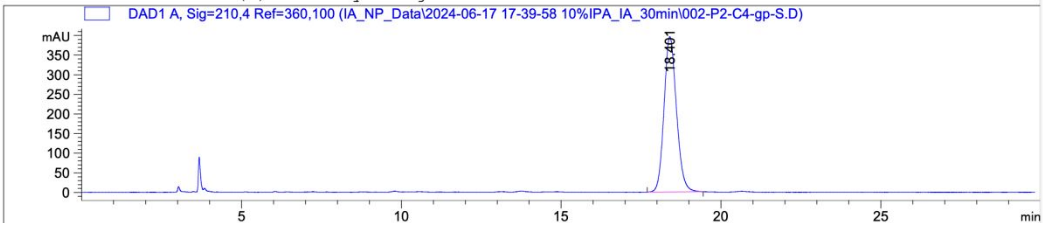


| Peak # | RetTime [min] | Type | Width [min] | Area [mAU*s] | Height [mAU] | Area % |
|---|---|---|---|---|---|---|
| 1 | 18.401 | VV R | 0.3194 | 1.06736e4 | 395.47723 | 100.0000 |

HPLC chromatogram of compound **35** (Chiralpak IA, 1 mL.min$^{-1}$, Hex/*i*-PrOH = 95:5)

```
Acq. Operator   : SYSTEM                          Seq. Line :   2
Sample Operator : SYSTEM
Acq. Instrument : Normal Phase                     Location :   P2-F-03
Injection Date  : 6/20/2024 1:46:08 AM                  Inj :   1
                                                 Inj Volume : 5.000 µl
Method          : C:\Users\Public\Documents\ChemStation\1\Data\IA_NP_Data\2024-06-20 01-34-44 10
                  %IPA_IA_30min\10_IPA_IA_FIXED_1.0mL-min_30min.M (Sequence Method)
Last changed    : 10/1/2022 2:18:02 PM by SYSTEM
Method Info     : Date Created: 09/27/2022
                  Created By: Patrick Gross
                  %IPA: 10
                  Column: IA
                  Flowrate: 1.0 mL/min
                  Runtime: 30.0 min
                  Wavelengths: 210nm, 230nm, 254nm, 280nm
```

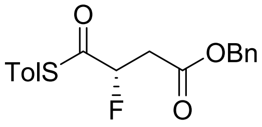


**35 (*S* isomer)**

```
Additional Info : Peak(s) manually integrated
```

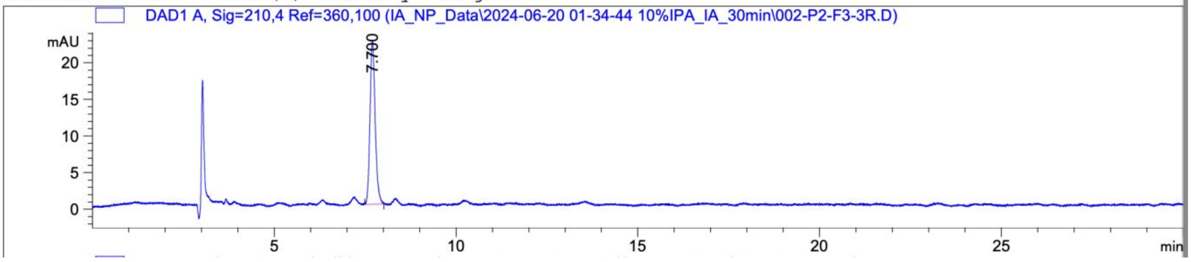


| Peak # | RetTime [min] | Type | Width [min] | Area [mAU*s] | Height [mAU] | Area % |
|---|---|---|---|---|---|---|
| 1 | 7.700 | VV R | 0.1172 | 220.20821 | 22.19733 | 100.0000 |

HPLC chromatogram of compound **13b** (Chiralpak IA, 1 mL.min$^{-1}$, Hex/*i*-PrOH = 95:5)

On top: mixture of (*R*) and (*S*) enantiomers; below: compound **13b**

```
Acq. Operator   : SYSTEM                          Seq. Line :   2
Sample Operator : SYSTEM
Acq. Instrument : Normal Phase                     Location :   P2-F-01
Injection Date  : 6/18/2024 7:14:36 PM                  Inj :   1
                                               Inj Volume : 5.000 µl
Method          : C:\Users\Public\Documents\ChemStation\1\Data\IA_NP_Data\2024-06-18 18-58-10 25
                  %IPA_IA_30min\25_IPA_IA_FIXED_1.0mL-min_30min.M (Sequence Method)
Last changed    : 10/1/2022 2:27:58 PM by SYSTEM
Method Info     : Date Created: 10/01/2022
                  Created By: Patrick Gross
                  %IPA: 25
                  Column: IA
                  Flowrate: 1.0 mL/min
                  Runtime: 30.0 min
                  Wavelengths: 210nm, 230nm, 254nm, 280nm

Additional Info : Peak(s) manually integrated
```

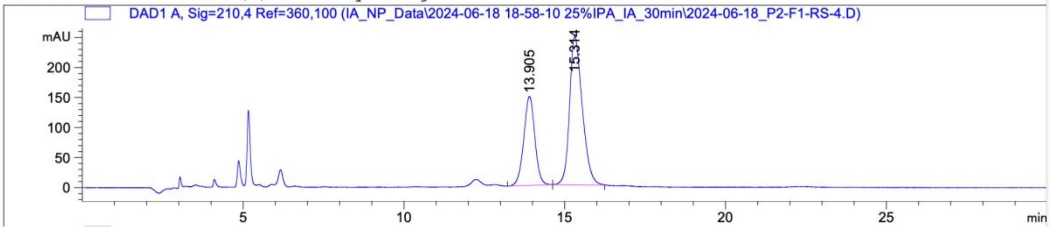


| Peak # | RetTime [min] | Type | Width [min] | Area [mAU*s] | Height [mAU] | Area % |
|---|---|---|---|---|---|---|
| 1 | 13.905 | VV R | 0.2818 | 3549.62012 | 148.28143 | 34.4411 |
| 2 | 15.314 | VV R | 0.3284 | 6756.74414 | 246.80832 | 65.5589 |

```
Acq. Operator   : SYSTEM                          Seq. Line :   2
Sample Operator : SYSTEM
Acq. Instrument : Normal Phase                     Location :   P2-F-02
Injection Date  : 6/18/2024 10:14:15 PM                 Inj :   1
                                               Inj Volume : 5.000 µl
Method          : C:\Users\Public\Documents\ChemStation\1\Data\IA_NP_Data\2024-06-18 21-57-50 25
                  %IPA_IA_30min\25_IPA_IA_FIXED_1.0mL-min_30min.M (Sequence Method)
Last changed    : 10/1/2022 2:27:58 PM by SYSTEM
Method Info     : Date Created: 10/01/2022
                  Created By: Patrick Gross
                  %IPA: 25
                  Column: IA
                  Flowrate: 1.0 mL/min
                  Runtime: 30.0 min
                  Wavelengths: 210nm, 230nm, 254nm, 280nm
```

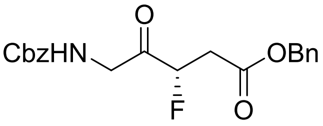


**13b (*S* isomer)**

```
Additional Info : Peak(s) manually integrated
```

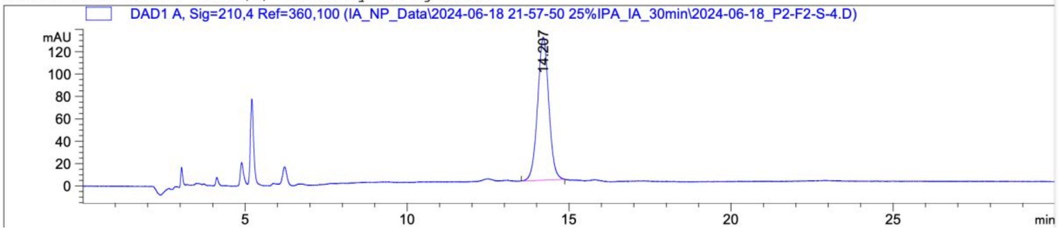


| Peak # | RetTime [min] | Type | Width [min] | Area [mAU*s] | Height [mAU] | Area % |
|---|---|---|---|---|---|---|
| 1 | 14.207 | VV R | 0.2856 | 3071.00830 | 127.93171 | 100.0000 |
